# Far-field, near-field and photothermal response of plasmonic twinned magnesium nanostructures†

**DOI:** 10.1039/d3nr05848d

**Published:** 2024-01-31

**Authors:** Christina Boukouvala, Claire A. West, Andrey Ten, Elizabeth Hopper, Quentin M. Ramasse, John S. Biggins, Emilie Ringe

**Affiliations:** a Department of Materials Science and Metallurgy, University of Cambridge 27 Charles Babbage Road Cambridge, CB3 0FS UK er407@cam.ac.uk; b Department of Earth Sciences Downing Street Cambridge, CB2 3EQ UK; c Department of Chemical Engineering and Biotechnology, University of Cambridge Philippa Fawcett Drive Cambridge CB3 0AS UK; d School of Chemical and Process Engineering, University of Leeds 211 Clarendon Road Leeds LS2 9JT UK; e School of Physics and Astronomy, University of Leeds Woodhouse Leeds LS2 9JS UK; f SuperSTEM, SciTech Daresbury Science and Innovation Campus Keckwick Lane Warrington WA4 4AD UK; g Department of Engineering, University of Cambridge Trumpington Street Cambridge CB2 1PZ UK

## Abstract

Magnesium nanoparticles offer an alternative plasmonic platform capable of resonances across the ultraviolet, visible and near-infrared. Crystalline magnesium nanoparticles display twinning on the (101̄1), (101̄2), (101̄3), and (112̄1) planes leading to concave folded shapes named tents, chairs, tacos, and kites, respectively. We use the Wulff-based Crystal Creator tool to expand the range of Mg crystal shapes with twinning over the known Mg twin planes, *i.e.*, (101̄*x*), *x* = 1, 2, 3 and (112̄*y*), *y* = 1, 2, 3, 4, and study the effects of relative facet expression on the resulting shapes. These shapes include both concave and convex structures, some of which have been experimentally observed. The resonant modes, far-field, and near-field optical responses of these unusual plasmonic shapes as well as their photothermal behaviour are reported, revealing the effects of folding angle and in-filling of the concave region. Significant differences exist between shapes, in particular regarding the maximum and average electric field enhancement. A maximum field enhancement (|*E*|/|*E*_0_|) of 184, comparable to that calculated for Au and Ag nanoparticles, was found at the tips of the (112̄4) kite. The presence of a 5 nm MgO shell is found to decrease the near-field enhancement by 67% to 90% depending on the shape, while it can increase the plasmon-induced temperature rise by up to 42%. Tip rounding on the otherwise sharp nanoparticle corners also significantly affects the maximum field enhancement. These results provide guidance for the design of enhancing and photothermal substrates for a variety of plasmonic applications across a wide spectral range.

## Abstract 2

This article explores the shapes and plasmonic properties of concave and convex magnesium nanoparticles, which hold promise for biocompatible and sustainable plasmonic applications.

## Graphical abstract

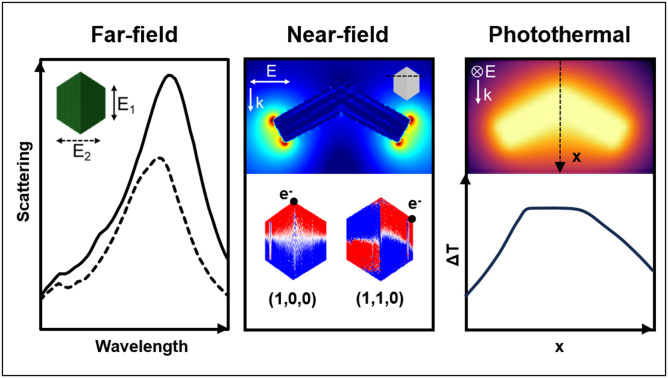

## 1. Introduction

The conduction electrons of nanoscale objects of some metals, notably Mg, Al, Cu, Ag, and Au, can be driven into resonance by the oscillating electromagnetic field of light, leading to localised surface plasmon resonances (LSPRs). LSPR properties, *e.g.*, absorption, scattering, resonance frequency, linewidth, and electric field localisation are observable in the near-field and far-field; they depend on nanoparticle (NP) composition, size,^1^ and shape,^2,3^ such that by tailoring fabrication or synthesis one can optimise performance in specific applications. For example, maximising absorption optimises photothermal heating,^4^ enhanced scattering can be used for sensing,^5^ and intense localised electric fields can be used to harness sunlight for photocatalysis^6^ and to enhance Raman scattering signals.^7^

Mg is a recent addition to the toolbox of plasmonic metals, which currently includes Au, Ag, Cu, and Al. Mg can sustain LSPRs across the ultraviolet-visible-near-infrared (UV-vis-NIR), and its plasmon quality factor, as dictated by its dielectric function, is higher than any of the aforementioned metals below 300 nm, second best after Ag up to ∼500 nm and higher than earth-abundant Al up to ∼900 nm.^8–10^ Mg's attractiveness, however, does not lie only on its competitive plasmon quality: it is biocompatible^10–13^ and significantly cheaper and more abundant than Au and Ag. Due to their constituent's high oxidation potential, Mg NPs form a protective self-limiting oxide layer^14^ that stabilises them in air, as opposed to the full oxidation observed in Cu NPs. Further, Mg crystallises in a hexagonal close packed (HCP) structure, different from the other plasmonic metals’ (Au, Ag, Al, Cu) face centre cubic (FCC) structure, leading to Mg's fundamentally different crystal shapes.

The potential applications of plasmonic NPs have fuelled interest in the effects of shape on plasmonic response, and, in turn, in shape control. Numerical and experimental shape studies span a range of single crystal and twinned FCC NP shapes such as cubes, bipyramids, rods, decahedra, and icosahedra^15–18^ of Au, Ag, and Cu as well as cubes, bipyramids, octahedra, icosahedra, and singly twinned shapes of Al.^19,20^ In contrast Mg features single crystal hexagonal prisms^14^ and singly twinned concave shapes with a V-shaped cross section.^21^ These unique folded geometries arise due to the twin planes possible in the HCP crystal structure.

NP shape models, such as the Wulff construction, can greatly facilitate structure–property investigations by providing input shapes for numerical calculations (*e.g.*, for solving Maxwell's equations) as well as an insight on NP growth mechanisms. The Wulff construction predicts the thermodynamic or kinetic shape of NPs by stating that the distance of a facet from the centre of the NP is proportional to its surface energy, or to the growth velocity in the kinetic case.^22,23^ Extensions to this theory have allowed the modelling of more complex systems. For instance, the modified Wulff construction^24^ accounts for the existence of twin planes in crystals by introducing one or more additional boundaries joining a parent crystal to one or more twins by a known relationship, in simple cases by a reflection. Additional growth parameters can be included in these models to mimic kinetic control and better match experimental shapes; for example, enhancement of growth along a defective twin plane can modify the NP's aspect ratio (AR), while the expected enhanced growth on concave facets (so-called re-entrant surfaces) leads to fully convex structures.^21,25^ Several implementations of these models are freely available and reviewed in ref. 26.

Single crystalline hexagonal prisms of Mg have been repeatedly synthesised^13,27–29^ and plasmonically characterised showing LSPRs spanning the UV-vis-NIR.^14^ Meanwhile, experimental evidence of singly twinned Mg NPs dates back to a 1981 gas phase evaporation study^30^ and was recently brought back to light by a colloidal synthesis.^21^ In the latter work, a HCP Wulff model incorporating twinning was developed to explain the observed NP shapes where twinning along the crystallographic planes (101̄1), (101̄2), (101̄3), and (112̄1) leads to folded shapes with varying folding angles named tents, chairs, tacos, and kites, respectively. The near-field and far-field optical response of some twinned Mg NPs were reported experimentally and numerically, leading to the identification of experimentally observed plasmonic modes and revealing the far-field effects of size, AR, substrate, and MgO layer.

The low symmetry and V-shaped cross section of these colloidally synthesised singly twinned Mg NPs is attractive as an unusual feature in colloidal plasmonic metals, enabled by the HCP lattice of Mg. Low symmetry structures reduce the mode degeneracy^31^ while V-shaped^32,33^ prismatic structures (also referred to as L-shaped for right angles^34–36^) have generated much interest as nano-antennas for polarisation conversion.^35,37,38^ The generation and investigation of the effects of such structural features has been limited so far by the common reliance on fabricated shapes, leading to flat or prismatic NPs bound to a substrate, or assemblies of, *e.g.*, colloidally synthesised nanorods.^39^ Mg NPs offer a further opportunity to probe near-field and far-field effects, including plasmonic light localisation and photothermal effects, as their shape is neither flat nor prismatic, in contrast to fabricated V- and L-shapes.

Here, we describe for the first time an extensive array of crystallographically possible shapes of singly twinned Mg NPs based on known bulk Mg twin planes^40,41^ and experimentally observed twinned NPs. In addition to the tent, chair, taco, and (112̄1) kite previously reported, in section 3.1 we expand the Mg shape set to include (112̄*y*), *y* = 2, 3, 4 twin planes. Further, we present their convex analogues, which occur with strong kinetic re-entrant enhancement and have been observed in gas-phase syntheses.^30^ In section 3.2, we explore the effects of these unusual shapes on the near-field and far-field plasmonic response with electromagnetic simulations and experimental electron-beam approaches. In section 3.3, we further explore shape effects by numerically comparing the optical properties of shapes with different folding angles, followed by section 3.4 which contrasts concave and convex structures. We then investigate how these differences can affect the application of plasmonic Mg NPs, first describing field enhancement (section 3.5) and finally photothermal effects (section 3.6) Together, these results enhance the understanding of plasmonic effects in shapes uniquely enabled by Mg's HCP lattice.

## 2. Materials and methods

### 2.1. Numerical methods

Shapes were obtained using the HCP Wulff construction function of Crystal Creator, a freely available crystal shape modelling tool.^42^ Optical scattering and absorption spectra were obtained numerically in the DDA using DDSCAT^43^ while electron excitation calculations were performed using a modified version of eDDA,^14,44^ itself a version of DDSCAT modified to replace the plane wave excitation with a stimulation of a swift electron beam. Thermal calculations were performed using t-DDA^45^ where the steady-state heat diffusion equation is solved on a discretised volume of thermally and electromagnetically coupled point dipoles. The temperatures were calculated at the longitudinal dipole resonance of each nanoparticle (*k*_Mg_ = 156 W m^−1^ K^−1^, *k*_MgO_ = 42 W m^−1^ K^−1^) in an infinite background of air (*k*_air_ = 0.003 W m^−1^ K^−1^) at laser intensity 10^7^ W m^−2^. In all DDA approaches, particles are represented by an array of small cubic elements, considered dipoles, interacting with each other and with the incident electric field. These interactions can be described by a system of Maxwell's equations that is solved to obtain the polarisation of each dipole and subsequently to calculate the far-field, near-field and photothermal properties of the particle.

For all calculations, the frequency dependent refractive index of metallic Mg was taken from Palik,^46^ the refractive index of MgO was set to 1.7, and the ambient refractive index was set to 1. To capture the shape anisotropy, a variety of different excitation and polarisation directions, all perpendicular to each other, were chosen. All far-field calculations were carried out with dipole distances (dd) between 1.2 nm and 1.7 nm, to ensure sufficient accuracy and convergence (Fig. S1†), such that the number of dipoles varies from ∼100 000 for concave to ∼420 000 for convex shapes. The convergence of the electric field enhancement requires a significant number of dipoles, as discussed in ref. 47, hence dd = 1 nm was used for mapping the electric fields and for better capturing shape modifications, *e.g.* rounding effects. Intervals of 10 nm and 0.1 eV were used for spectral calculations in DDA and eDDA, respectively. Scattering and extinction efficiencies, taken directly from DDSCAT output, are given as *C*_sca_/(π*α*_eff_^2^) and *C*_ext_/(π*α*_eff_^2^) respectively, where *C*_sca_ is the scattering cross section, *C*_ext_, the extinction cross section, and *α*_eff_, the radius corresponding to a sphere of equal volume. Near-field enhancements are reported as obtained from DDA, *i.e.* as the enhancement factor of the root-mean-square amplitude of the electric field. Positive and negative charge distribution (in arbitrary units) was calculated at distance dd from the NP surface as the dot product of the electric field with the surface normal at each point.

### 2.2. Synthesis and characterisation

Magnesium nanoparticles were synthesised by the colloidal reduction of di-*n*-butylmagnesium by lithium naphthalenide, as previously reported.^48^ Low-loss STEM-EEL spectra of Mg NPs drop cast on a 10 nm thick Si_3_N_4_ membrane (Simpore Inc.) were obtained on a Nion UltraSTEM™ 100MC High Energy Resolution Monochromated STEM-EELS (HERMES) microscope, a dedicated STEM equipped with a cold field electron emitter, and Nion's ultrahigh resolution ground-potential monochromator. The microscope was operated at 60 kV, with an electron probe of 31 mrad convergence semi-angle and current of 50 pA before closing the monochromator slit, corresponding to a probe size of approximately 1 Å. EEL spectra were recorded on a Nion Iris spectrometer, equipped with a Dectris ELA hybrid pixel detector camera, with a 44 mrad semi-angle entrance aperture and an angular range of 90–195 mrad for the high-angle annular dark field (HAADF) imaging detector. The system's monochromator slit size choice for these experiments resulted in a 75 meV full width at half-maximum of the zero-loss peak and 5–7 pA current. For every spectrum, the energy axis was aligned with subpixel accuracy using the zero-loss peak before denoising using principal component analysis on the Gatan Digital Micrograph software. The resulting EEL spectral data was processed using an open-source software, Hyperspy.^49^ Spectra were cropped from 0.30 eV to 7.00 eV, after which spikes were removed and the minimum intensity was shifted to 0. The Mg bulk plasmon map was obtained by integrating the spectrum between 9.0 and 11.0 eV, the region around the bulk plasmon peak (∼9.9 eV). LSP modes were extracted using non-negative matrix factorisation (NMF)^3^ and fitted with a Lorentzian line shape; the number of NMF components was optimised for individual NPs, by selecting the largest value which did not cause the duplicate factorisation of identical modes.

## 3. Results and discussion

### 3.1. Twinned Mg NP shapes

**Fig. 1:**
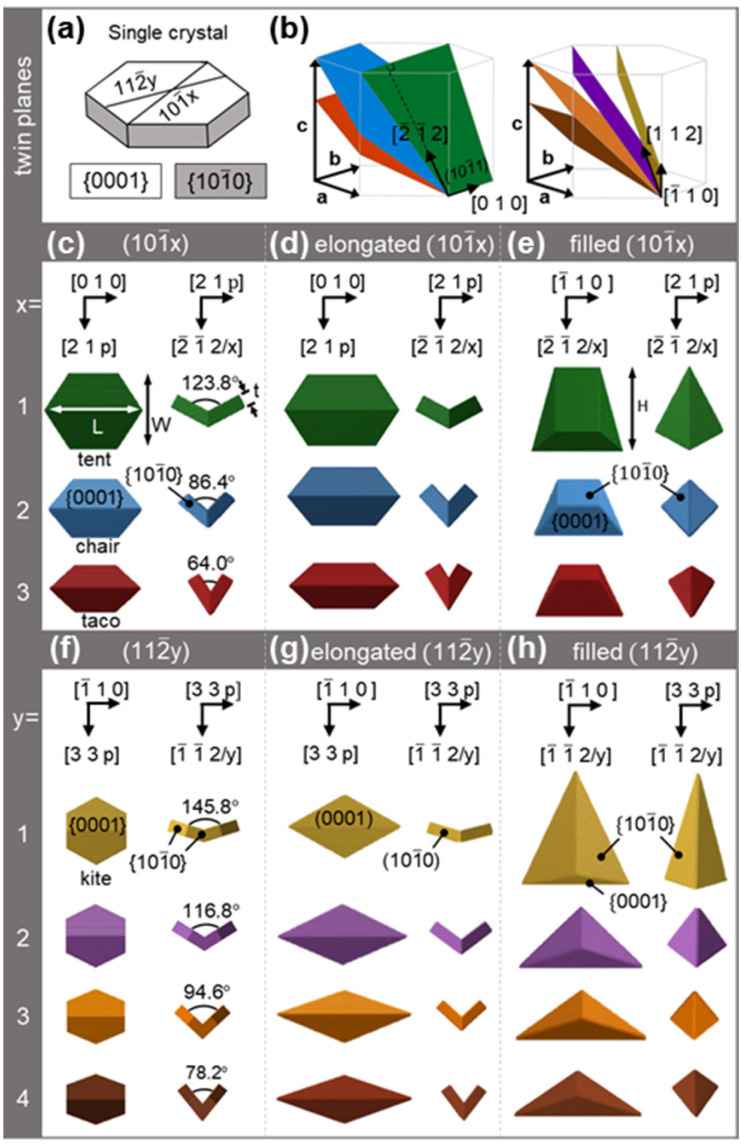
Twinned Mg crystal shape models. (a) Single crystal Mg hexagonal plate with colour coded prismatic {101̄0} and basal {0001} facets. (b) The two sets of Mg twin planes, where the twin planes (101̄*x*), *x* = 1, 2, 3 (left) and (112̄*y*), *y* = 1, 2, 3, 4 (right) are shown in green, blue, red, yellow, purple, orange and brown, respectively. (c), (d), (f), and (g) views along the vertical and longitudinal axis of concave shapes and (e) and (h) along the transverse and longitudinal axis for the filled shapes. Shapes are colour-coded based on their twin plane and viewing directions are as indicated, with *p* = 1.5*xa*^2^/*c*^2^ or 1.5*ya*^2^/*c*^2^ for type I and type II twins, respectively, where a and c are the lattice vectors. Directions are consistent with the lattice vectors in (b) and correspond to either the bottom or left twin of the twinned structures when both crystals are visible. Arrows on the (101̄1) twinned NPs (tent and filled tent) in (c) and (e) indicate the length (*L*), width (*W*), thickness (*t*), and height (*H*).

Unlike FCC metals (Au, Ag, Cu, Al, *etc.*), Mg crystallises in HCP which supports many twin planes and thus leads to a large number of thermodynamic and kinetic shapes (Fig. 1 and S2, S3†). The possible twin planes consist of three first-order pyramidal crystallographic planes (101̄*x*), *x* = 1, 2, 3 (pyramidal I, Fig. 1b left) leading to type I twins (Fig. 1c–e and S2b, e†), and four second-order pyramidal crystallographic planes (112̄*y*), *y* = 1, 2, 3, 4 (pyramidal II, Fig. 1b right) leading to type II twins (Fig. 1f–h and S2c, f†). When using relative growth velocities consistent with that of experimentally observed hexagonal prisms,^13,27,28^ these twinned shapes appear as folded hexagonal prisms displaying mirror symmetry along the central twin plane (Fig. 1c, f and S2e–f†).

In type I twins, the crystallographic orientation relationship between the parent (*α*) and twin (*β*) crystals can be formally described as (101̄*x*)_*α*_||(101̄*x*)_*β*_, [01̄10]_*α*_||[011̄0]_*β*_. The (101̄1), (101̄2), and (101̄3) twin planes form angles of 61.9°, 43.2°, and 32.0°, respectively, with the basal (0001) plane (Fig. 1b); the inner folding angle is twice this angular value (Fig. 1c). We used these relationships, together with the lattice parameters of Mg (*a* = 3.19 Å and *c* = 5.18 Å (ref. 50)), to model crystallographically correct structures in our Wulff-based software, Crystal Creator.^42,51^ Thermodynamic and kinetic shapes yield small {101̄1} re-entrant notches (Fig. S2b, e and S3†) that are not observed experimentally. Sharp type I twin shapes better matched to synthesis results are instead obtained by adding small amounts of kinetic growth enhancement (parameters in Table S1†) representing the preferential growth occurring at these notches, as previously done for FCC metals.^25^ Imposing an additional kinetic effect, namely growth enhancement along the twin plane, elongates the structures (Fig. 1d). Both regular and elongated type I twins have been reported previously,^21^ although what controls their experimental occurrence ratio remains unclear.

In the type II twins, which we also call kites, the orientation relationship is (112̄*y*)_*α*_||(112̄*y*)_*β*_, [1̄21̄0]_*α*_||[12̄10]_*β*_. The (112̄1), (112̄2), (112̄3), and (112̄4) twin planes (Fig. 1b) form angles of 72.9°, 58.4°, 47.3°, and 39.1°, respectively, with the basal (0001) plane, leading to folding angles of 145.8°, 116.8°, 94.6°, and 78.2°. This twinning (Fig. 1f and S2f†) results in truncated kites, that turn into kite shapes (Fig. 1g) when kinetic twin growth enhancement is applied. Of these many shapes, only the (112̄1) kite of the sharp kind has been conclusively reported experimentally,^21,30^ such that we will focus on the sharp kites (Fig. 1f) for the remainder of the paper.

Filled, and therefore convex, type I and type II structures were also modelled, based on experimental results. Ohno and Yamauchi^30^ observed filled structures related to tents, tacos, and (112̄2) truncated kites while Asselin, Boukouvala *et al.*^21^ suggested filled chairs. More recently Ghildiyal *et al.*^52^ have also reported shapes resembling filled tents. These structures can be obtained numerically by considering a kinetic growth where the central concave fold offers a favourable growth site, leading to in-filling of the inner (0001) facets, eventually resulting in a convex shape (Fig. 1e and h).

All shapes expose the lowest surface energy facets, *i.e.*, the basal {0001} and the prismatic {101̄0} planes, as expected from the numerical parameters chosen. They also all feature two symmetry planes, one coincident with, and the other perpendicular to, the twin plane. Type I and type II twins feature six and four {101̄0} facets, respectively, and both sets of shapes have four {0001} facets. Meanwhile, convex shapes have extended {101̄0} facets, six for filled type I twins and four for filled kites, and only two {0001} exposed facets.

For discussion purposes, we define size parameters (length *L*, width *W*, thickness *t*, height *H*) for the above-mentioned structures as labelled in Fig. 1c and e. Briefly, *L* is the tip-to-tip distance on the intersection of the twin and (0001) planes (longitudinal or long NP axis), *W* is the maximum dimension perpendicular to the twin plane (transverse or short NP axis), and *t* is the thickness of each individual crystal (sometimes referred to as the NP “wing” in contrast to the NP “arm” terminology used for flat structures^32,35,38^). For filled shapes, *t* is replaced by *H*, the total dimension along the third NP axis (vertical axis), which is perpendicular to both the short and long NP axes. The aspect ratio (AR) is defined as *L*/*W*.

### 3.2. Shape effects on plasmon modes

Composition, size, and shape play a key role in dictating the plasmonic properties of NPs. Interest in using Mg for plasmonics stems from its abundance, biocompatibility, and resonance range; its plasmonic resonances are slightly broader than for Ag and Au for the same shape, but of comparable intensity, as shown in Fig. S4.† Straightforward size effects such as a redshift of the LSPRs with increased length and AR have already been revealed for twinned Mg shapes such as tents in a previous publication.^21^ These well-known relationships also apply to all the twinned shapes here. The shape effects are however more interesting and unusual due to the unique folded geometry of both type I and type II twins, and the subtle interplay of size across all three NP dimensions. To investigate these effects on the plasmonic modes, Maxwell's equations were solved in DDA for both photon^43^ and electron excitation^44^ for four representative shapes of the same *L* (200 nm): a tent (*W* = 158 nm, *t* = 28 nm), a filled tent (*W* = 158 nm, *H* = 190 nm), a (112̄1) kite (*W* = 111 nm, *t* = 28 nm), and a filled (112̄1) kite (*W* = 111 nm, *H* = 213 nm); the latter two will subsequently be referred to simply as kite and filled kite, respectively.

**Fig. 2:**
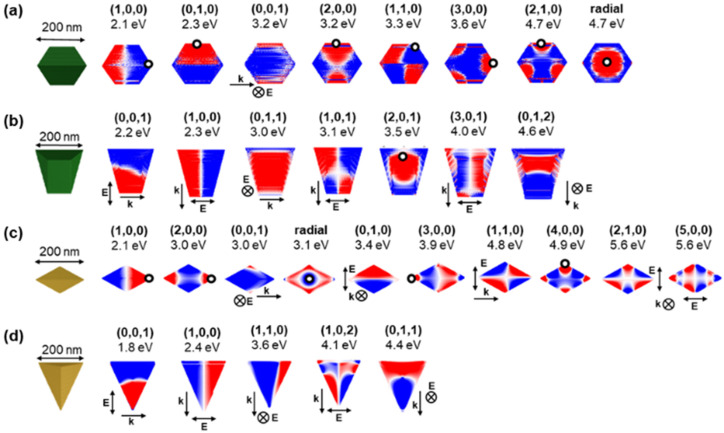
Calculated near-field charge distribution of resonant modes in tent and kite shapes of Mg NPs. Plasmon modes of (a) tent, (b) filled tent, (c) (112̄1) kite and (d) filled (112̄1) kite where blue and red colours (arbitrary intensity) correspond to opposite charges on the NP's surface. Electron beam excitation is denoted by a black circle at the beam position, with propagation perpendicular to the page, while for photon excitation, the incident field direction (***k***, single arrow) and polarisation (***E***, double arrow) are shown. Incident field directions and polarisations perpendicular to the page are denoted with ⊗ and a ***k*** or ***E*** annotation.

The dominant LSPR modes of the four shapes are shown in Fig. 2, S5 and S6.† The first notable observation is that given the anisotropic geometry of the NPs, with *L* ≠ *W ≠ t* or *H*, the charge distribution can be rather complex and is not evidently describable by a “dipole” and “quadrupole”-type nomenclature. Also, the interpretability of the charge distribution is slightly obscured by retardation effects, present because of the rather large (experimentally relevant) NP size chosen in the numerical modelling. Nevertheless, we succeeded at assigning the observed modes and chose to use a three index system (*l*,*w*,*h*), denoting the number of nodes across the longitudinal, transverse and vertical axes of the NP.^21^

The modes of a tent shaped NP are shown in Fig. 2a and S5a.† Three dipole modes appear at low energies; a (1,0,0) or longitudinal dipole at 2.1 eV, a (0,1,0) or transverse dipole at 2.3 eV as well as a weak (0,0,1) mode at 3.2 eV. The latter is equivalent to the first symmetric (or antibonding) mode for flat V-shaped structures as opposed to the (0,1,0) being often called the antisymmetric (or bonding) mode.^32,33,39^ These are followed by the (2,0,0) and (1,1,0) modes appearing at very close energies (3.2 eV and 3.3 eV), as well as a (3,0,0) and (2,1,0) mode (see also Fig. S5c and ESI† for a more detailed mode description) at 3.6 eV and 4.7 eV, respectively. Finally, at 4.7 eV, a breathing mode, *i.e.*, a mode with radial symmetry for which the (*l*,*w*,*h*) notation is poorly suited, can be excited when the electron beam is travelling through the center of the NP. Note that while e-DDA does not take into account changes in the propagation of the beam within the NP,^44^ it nevertheless produces an interpretable and realistic mode symmetry and corresponding resonance energies.

The kite shape (Fig. 2c) has a higher AR than the tent shape such that a rearrangement of the relative energy of the modes occurs, with high l modes appearing at lower energies. For example, the (2,0,0) mode is at 3.0 eV, *i.e.*, immediately following the 2.1 eV (1,0,0) mode; another example is the (3,0,0) mode, which now resonates at 3.9 eV, before the (1,1,0) mode now at 4.8 eV. Further to this mode reshuffling, additional modes, not present in the tent shape, appear. At 4.9 eV, a mode with nodes at each tip is observed with an overall charge distribution very similar to (4,0,0). Further, a (5,0,0) mode emerges at 5.6 eV, reinforcing the more rod-like nature (higher AR) of the kite *vs.* the tent. Similarly to the tent having modes related to those of a hexagonal prism,^14^ the kite has modes comparable to those of the rhombohedral prism described by West *et al.*,^53^ such as a longitudinal and transverse dipole as well as a quadrupole mode.

Filling of the NP drastically changes its mode charge distribution and energy (Fig. 2b and d). The first two low energy modes in both the filled kite and filled tent correspond to the two dipoles, (0,0,1) and (1,0,0), however, in contrast to the concave shapes, the transverse (0,1,0) dipole is vanishingly weak (Fig. S6†). This is because *L* and *H* are both much larger than *W*. The low energy (0,0,1) dipole appears at lower energy for the filled kite (1.8 eV) than that for the filled tent (2.2 eV) owing to the kite's larger *H* (23 nm higher) and more tapered, *i.e.*, more anisotropic, shape. Higher energy modes have multiple nodes often in different orientations than those of the concave structures, again due to their large *H* when compared to *t*. For instance, the filled shapes both sustain a strong (0,1,1) mode at the relatively low energy of 3.0 eV (filled tent) and 4.4 eV (filled kite). Several further modes are observed at higher energy as shown in Fig. 2b and d.

**Fig. 3:**
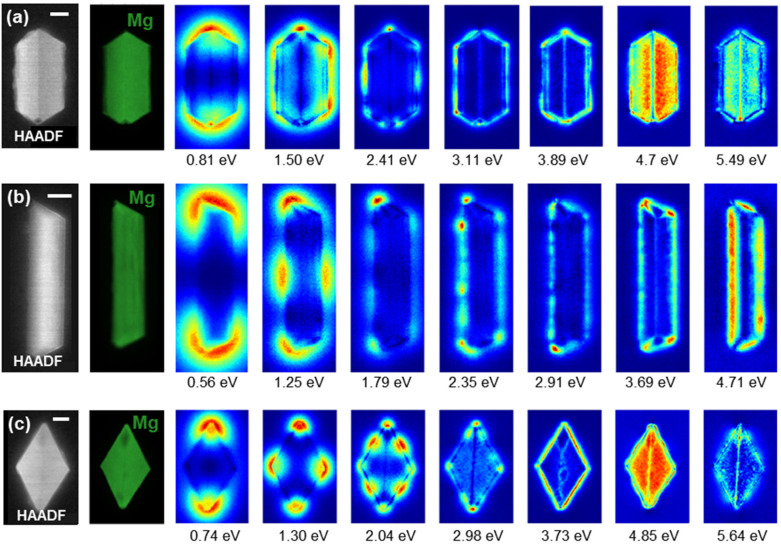
Experimental map of plasmon excitation probability. STEM-HAADF (left), Mg bulk plasmon maps (in green), and low-loss STEM-EELS NMF spatial loadings, with peak energies indicated in the figure for (a) *L* = 475 nm tent, (b) *L* = 792 taco, and (c) *L* = 643 nm (112̄1) kite Mg NPs. Spectral components are reported in Fig. S7.† Scale bars, 50 nm.

The shape-dependent plasmon modes can also be experimentally revealed *via* mapping of the plasmon excitation probability with an electron beam performed in the UV-vis-NIR range using monochromated STEM-EELS. Excitation maps for colloidally synthesised tent, taco, and (112̄1) kite shape, shown in Fig. 3, reveal experimental modes with excitation geometries that correspond well to those expected from the surface charges in Fig. 2; note that the distributions are not the same between Fig. 2 and Fig. 3, as the first represents charge distribution, and the second, loss probability.^14,21^ However, the experimental modes are observed at different energies due to size, MgO presence and AR differences. An upper bound to the MgO thickness of the synthesised Mg NPs was found to be 10–15 nm based on the STEM-HAADF and bulk plasmon maps; this value agrees with what has been previously reported for colloidal syntheses,^21^ and is larger than the 3–5 nm reported for gas-phase syntheses.^60,61^

Experimentally (Fig. 3), the (1,0,0) mode for the tent shape (*L* = 475 nm, AR = 1.94) appears at 0.81 eV, at lower energy than the shorter simulated tent in Fig. 2 (*L* = 200 nm, AR = 1.27), as expected. The dipole is followed by higher order modes in an order slightly modified from the numerical calculations but following that of previously reported tents of similar AR.^21^ Some modes are not clearly visible, for instance (0,1,0) which is weak and (2,0,0) which is very close or overlapping with (1,1,0) (ref. 21 and Fig. S5a†). At high energy, modes are also not clearly attributable as several excitations accumulate and overlap. The high AR of the taco shape (*L* = 792 nm, AR = 4.6) shifts the modes with multiple nodes along the NP length to lower energies compared to those of the calculated shape, with the first three modes corresponding to (1,0,0) at 0.56 eV, (2,0,0) at 1.25 eV, and (3,0,0) at 1.79 eV. Higher energy modes display an increasing number of nodes. Finally, kite shapes maintain the same aspect ratio due to their crystallographic makeup; hence we do not expect significant mode reshuffling for the (112̄1) kite in Fig. 3 (*L* = 643 nm, AR = 1.71) as compared to simulations. The following modes can indeed be identified in the same order of appearance as in the simulations: (1,0,0) at 0.74 eV, (2,0,0) at 1.30 eV, (1,1,0) and (4,0,0) overlapping at 2.04 eV, followed by higher order modes.

### 3.3. Influence of folding angle on field localisation

The folding angle of the twinned Mg NPs is determined by their twin plane; for instance, the folding angle decreases from the tent to the chair and taco shape. For constant *L* (200 nm) and *t* (28 nm), changing twin plane leads to a change in the cross section and the distance between the side edges (for type-I twins) or tips (for type-II twins). These geometric differences are at least partially encoded in the value of the AR, which is 1.27, 1.58, and 1.95 for the tent, chair, and taco and 1.78, 1.99, 2.36, and 2.70 for the *y* = 1, 2, 3, and 4 kites, respectively. To understand the more complex relationships arising due to the V-shaped cross section, some average dimension of the NP could also be introduced; for instance the shape functional used by Sukharev *et al.*^38^ to explain geometric effects on the LSPRs of Ag L-shaped flat structures, *i.e.*, with similar cross section to the chair shape.

**Fig. 4:**
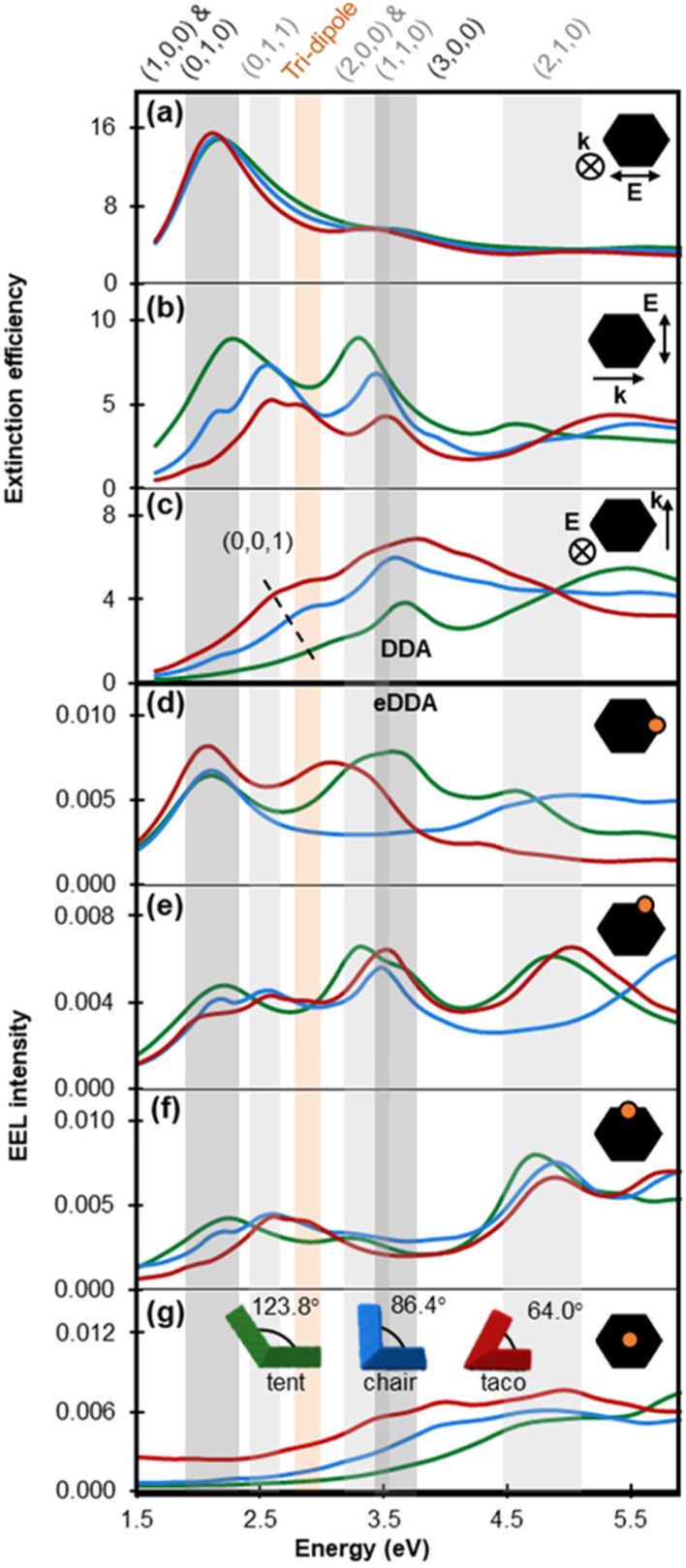
Numerically obtained plasmonic response and dominant modes of *L* = 200 nm tent (green lines), chair (blue lines) and taco (red lines) shapes. (a–c) Calculated extinction efficiency obtained from an optical excitation for various light propagation directions (single arrow) and polarisation directions (double arrow). Out of plane light propagation and polarisation directions are indicated with ⊗. (d–g) Calculated EEL spectra obtained with an electron beam excitation travelling out of plane for various beam positions, as shown by an orange dot on the projection of a tent shape (black); the electron beam excitation position is equivalent for each shape.

The changes in geometric features, including AR, have a higher impact at the resonant energy of modes exhibiting transverse and vertical nodes. Fig. 4 shows the calculated extinction and EEL spectra for the tent, chair and taco shapes; bands indicate the energy range over which one or more modes appear for the three shapes. The calculated extinction is dominated by the NPs’ scattering for longitudinal and transverse polarisation, while scattering and absorption contribute almost equally to the extinction cross section for vertical polarisation (Fig. S8†). Modes with nodes only along the longitudinal direction depend mainly on the NP length and are not expected to vary significantly with AR and folding. Indeed, (1,0,0) and (0,1,0) shift less than 30 nm as the folding angle decreases while the (2,0,0) appears in Fig. 4f as a well-defined peak only for the tent shape; it becomes a weak shoulder for the chair and disappears for the taco.

The (0,1,0), a transverse dipole, appears clearly for light polarised along the width of the NP (Fig. 4b) or an incident electron beam along its side (Fig. 4e–f). Its energy is close to that of the longitudinal mode for the tent shape because of the tent's low AR of 1.27. The (0,1,0) mode becomes weaker and redshifts as the folding angle decreases in accordance to what has been reported for the antisymmetric mode of flat V-shaped structures.^32,33^ A different trend emerges for the (0,0,1) mode (dashed line in Fig. 4c): as the NP wings gradually align with the vertical polarisation direction, the dimension along which electrons oscillate lengthens, and therefore the LSPR wavelength as well as both extinction efficiency and cross section increase. The intensity increase for (0,0,1) follows the same trend as the symmetric mode reported for V-shaped antennas of decreasing V angle however the peak shifts in the opposite direction,^32,33^ a difference that can be attributed to the three-dimensional nature of the structures studied here.

The modes beyond dipoles are also affected by the folding angle. For instance, the (0,1,1) mode becomes pronounced in chair and taco shapes due to the gradual alignment of the NP wings, resulting in the appearance of a low energy *h* = 1 node (Fig. 4b). This mode occurs at 2.6 eV for both chair and taco shapes. Interestingly, in taco shapes another mode appears at 2.9 eV. To clarify the nature of this mode its charge distribution for different phases of the excitation field were plotted (Fig. S9†). We learn that this mode lacks a node along the length of the NP so it cannot be called a (1,1,1). Instead, the charge distributions suggest that the interaction between the two NP wings forms a type of nanocavity, similar to what has been reported by Husu *et al.* for L-shaped NPs^35^ and Liu *et al.* for plano-semi-cylindrical nanocavities.^54^ In the latter, and thus here (Fig. 4), this mode is called a “tri-dipole” because of the existence of three dipole interactions within the cavity. Finally, the (1,1,0) (Fig. 4b) and (2,1,0) (Fig. 4f) modes both blueshift as the folding angle decreases.

**Fig. 5:**
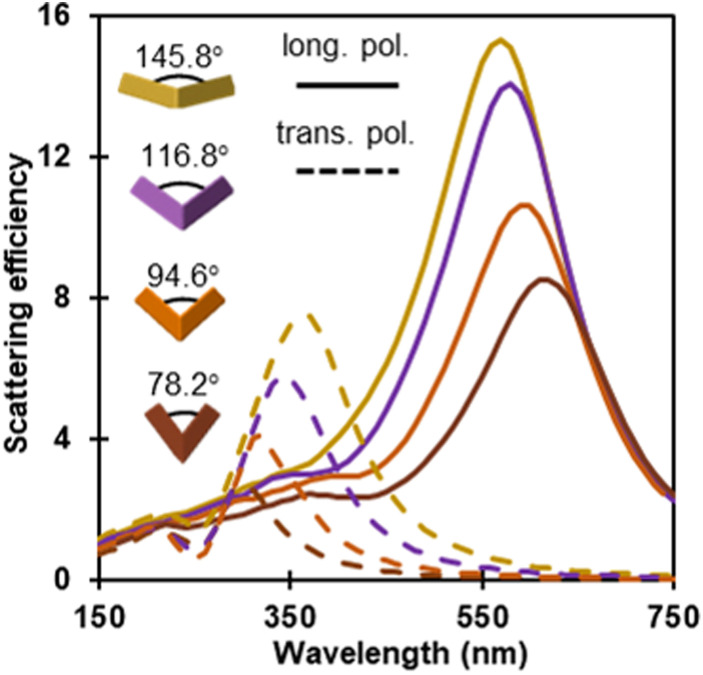
Calculated extinction efficiency for longitudinal (solid line) and transverse (dashed line) polarisation for the four kite shapes, modelled for kites of the same length (L = 200 nm).

Kite shapes exhibit ARs ranging from 1.78 to 2.7 and the scattering profile of 200 nm long kites (Fig. 5) demonstrates a clear AR effect. For instance, the (1,0,0) longitudinal dipole redshifts and the (0,1,0) transverse dipole (potentially overlapping with a (0,1,1) and/or tri-dipole modes) blueshifts as the folding angle increases. This trend agrees well with what is observed for nanorods.^2^ Additionally, as the folding angle decreases from 145.80° to 78.2°, the extinction efficiency at the peak wavelength drops with the exception of the (112̄2) kite; this is because of the combined effect of the increase in the absorption cross section and the decrease in the scattering cross section as the folding angle, and hence the volume, decreases (Fig. S10†).

As the shapes under consideration are often obtained in colloidal form, we also report in Fig. S11† orientation averaged extinction cross section spectra which are proportional to the experimental observable of absorbance. The multitude of LSPRs for both concave and convex structures results in three distinct peaks for the former and significantly overlapping LSPRs for the latter.

### 3.4. From concave to convex shapes

**Fig. 6:**
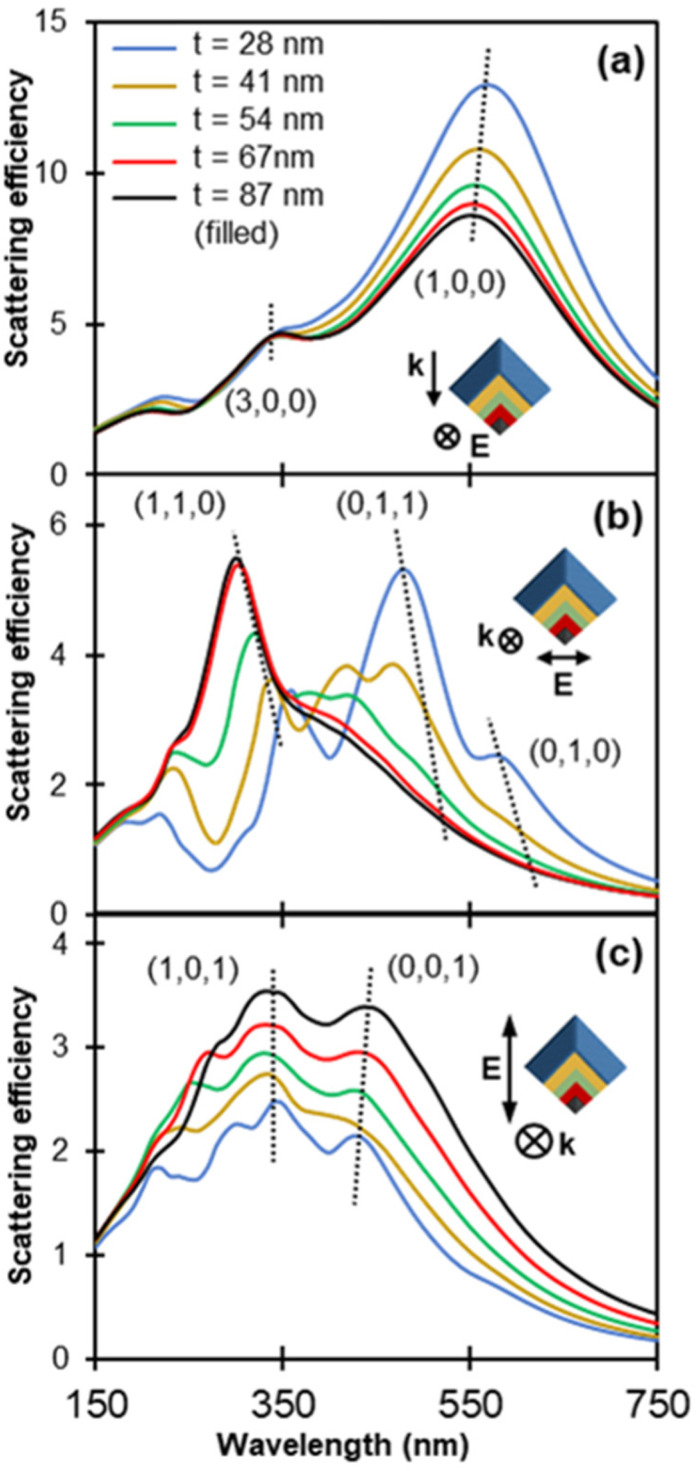
Calculated scattering spectra obtained for an optical excitation for progressively filled chair shaped particles (*L* = 200 nm, *W* = 127 nm) for (a) longitudinal, (b) transverse, and (c) vertical polarisation. Insets illustrate the NPs’ cross section overlaid on top of each other such that their thickness increases in the order of blue (thinnest chair), yellow, green, red and black (filled chair). Spectra are colour coded accordingly and the legend indicates the thickness. Polarisation directions and light propagation directions are indicated with double and single arrows, respectively, and with the symbol ⊗ when out of plane.

The changes in the scattering efficiency for three different polarisations for a geometric progression from a concave chair to a convex filled chair shape are shown in Fig. 6 and S12, 13.† As expected, the longitudinal dipole (1,0,0) is quite insensitive to the cavity filling, as its resonance energy predominantly depends on the length of the particle; only a small blueshift (∼10 nm) is observed from the thinnest concave to the fully convex chair (Fig. 6a). No significant shift is detected for the (3,0,0) mode. These observations are consistent with the slight blueshift reported previously for Mg tent shapes^21^ and for Au nanodisks^55^ of increasing thickness and can be explained by the decrease in shape anisotropy.^15^

The transverse dipole, (0,1,0), redshifts with filling as shown in Fig. 6b and S12b, 13.† This is in contrast to the blueshift previously reported for flat L-shaped structures of increasing thickness.^38^ The (0,1,1) mode exhibits a larger redshift of at least 60 nm while the high intensity (1,1,0) peak blueshifts. Additional peaks characterised by (*l*,*w*) = (1,1) also redshift (Fig. S13†), and their prevalence varies as the structure gets filled, a behaviour that could arise due to interference effects.

LSPRs do not shift appreciably upon filling for modes excited with a vertical polarisation, *i.e.*, for modes involving a node along the *t* or *H* dimension. For instance, only a small redshift of ∼10 nm is observed for the (0,0,1) mode as the chair goes from thin to filled. This redshift is in agreement with the work of Sukharev *et al.*^38^ that shows a redshift of the vertical dipole, albeit above a certain thickness. Additionally, the higher order mode at ∼300 nm disappears as the shape gets filled and hence the distance between the tips increases, indicating a relation to a tip-to-tip interaction, as also observed by Sukharev *et al.*^38^

However, the structures we study here are not just extruded shapes but have features in all three dimensions. Consequently, filling a chair shape does not produce the four-fold symmetry object reached by Sukharev *et al.*, and the chair shape's cross-sectional AR diverges from 1 as we move along the longitudinal axis towards the NP tips. Therefore, the transverse and vertical dipoles do not eventually coincide in filled chairs (Fig. S14†). We can also contrast the three-dimensional anisotropic structure of a filled chair with the simple behaviour of a square cross section beam (an extruded shape). Shokova *et al.*^56^ have shown numerically that such beams can support a longitudinal dipole (1,0,0) and a longitudinal quadrupole (1,1,0), or equivalently in their case (1,0,1), for longitudinal polarisation and light propagation along the short NP axis. In the same fashion, a transverse dipole (0,1,0) and transverse quadrupole (0,1,1) exist for transverse polarisation and light propagation also along the short NP axis. In the case of the filled chairs and for longitudinal polarisation, instead of a longitudinal quadrupole, (1,1,0), we observe a (3,0,0) mode (Fig. 6a) due to the sharp NP tips that localise charges. A (1,1,0) mode can instead be excited by transverse polarisation and light propagation direction along the long NP axis (Fig. 6b).

Lastly, scattering intensities are affected by the filling of chair shapes. Fig. 6 shows the scattering efficiency, *i.e.*, the scattering cross section divided by the area of the equatorial disk of an equivolume sphere, while plots for the scattering cross section are reported in Fig. S12.† The scattering efficiency of the longitudinal dipole (1,0,0) drops by 30% as the volume increases while that of the (3,0,0) mode remains constant. The scattering cross section remains almost the same for (1,0,0) hence the decrease in efficiency for the dipole is attributed to the increased volume while the absolute scattering for (3,0,0) is volume independent. For transverse polarisation, the (0,1,0) scattering efficiency decreases and eventually the peak vanishes for thicker structures. This is consistent for both light propagation directions, namely along the long axis (Fig. 6b) and the vertical axis (Fig. S12d†). Of the modes excited for transverse polarisation, the (1,1,0) for the filled shape exhibits the highest efficiency. Contrary to longitudinal polarisation, a significant increase is observed in the scattering efficiency (Fig. 6c) and the scattering cross section (Fig. S12c†) for vertical polarisations as the chair shape gets filled.

### 3.5. Electric field enhancement

**Fig. 7:**
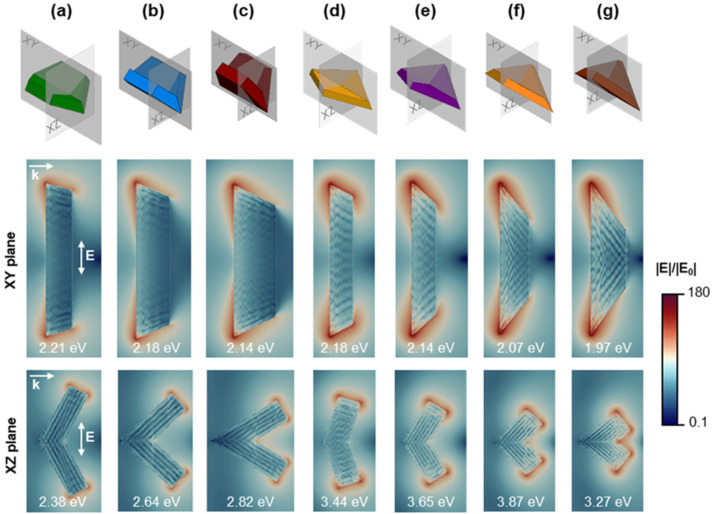
Calculated electric field enhancement |*E*|/|*E*_0_| plotted in logarithmic color scale for two cross sections of the (a) (101̄1) tent, (b) (101̄2) chair, (c) (101̄3) taco, (d) (112̄1) kite, (e) (112̄2) kite, (f) (112̄3) kite, and (g) (112̄4) kite. The field enhancement was calculated at the highest intensity far-field extinction peak for longitudinal polarisation in the *XY* plane and transverse polarisation in the *XZ* plane. NP length, *L* = 200 nm.

The unusual three-dimensional concave morphology of Mg NPs prompts the exploration of their localised electric field enhancement, an indication of the NPs’ potential for surface enhancement for applications in spectroscopies such as surface enhanced Raman spectroscopy and metal enhanced fluorescence. The field enhancement, |*E*|/|*E*_0_| is shown in logarithmic colour scale in Fig. 7 for the type I twins and the kites. Field enhancement is calculated at the most intense LSP energy for both longitudinal and transverse polarisations, while the cross sections are chosen such that they include the maximum enhancement regions, *i.e.*, the NP corners. Maximum and average enhancement values are reported in Table S2† for all the calculated shapes and for both longitudinal and transverse polarisations. Although the maximum near-field enhancement position is redshifted compared to the far-field peak intensities due to damping effects,^57^ we choose to show near-field enhancement at the peak of the extinction spectrum to mimic the experimental reality where peaks are determined from far-field observations. We calculate, for completeness, that the magnitude of this redshift is ∼50 nm for a tent and with a modest increase in |*E*|/|*E*_0_|_max_ of 8.7%, while for a (112̄4) kite (at the wavelength resolution of our calculations) the extinction and |*E*|/|*E*_0_|_max_ peaks overlap (Fig. S15a and c†). Note that this is due to the kite's smaller volume in accordance with previous reports.^57^ These small variations do not affect our conclusions related to the shape-dependence of the enhancement.

For type I twins, the maximum field enhancement for longitudinal polarisation is found on the twin plane cross section (*XY* plane) while for a transverse polarisation, it is seen on a plane perpendicular to the twin plane (*XZ* plane) and including a set of corners (Fig. 7a–c). For longitudinal polarisation, the maximum field enhancement increases significantly from 39.9 to 63.0 and 79.4 for the tent, chair, and taco shapes, respectively. As expected, this is proportional to the sharpness of the tips which increases in the same order, with tip angles of 115°, 100°, and 85°, respectively. The average enhancement on the NP surface is an order of magnitude less than the maximum (*e.g.*, tent |*E*|/|*E*_0_|_av_ = 3.03) but follows the same trend, *i.e.*, increases with increasing tip sharpness. However, the change between the shapes is less drastic; for the taco, the average field enhancement is only ∼20% higher than that of the tent. For transverse polarisation, the enhancement peaks at the cross-section's corners for the type I twins. The maximum enhancement values for all three shapes are very close, all falling between 25.6 and 27.9, noticeably lower than enhancements for longitudinal polarisation. As the folding angle decreases and the tips (and edges) are brought closer together, the enhancement within the concave angle increases; this results in the taco shape having a slightly higher average enhancement than the other shapes.

**Fig. 8:**
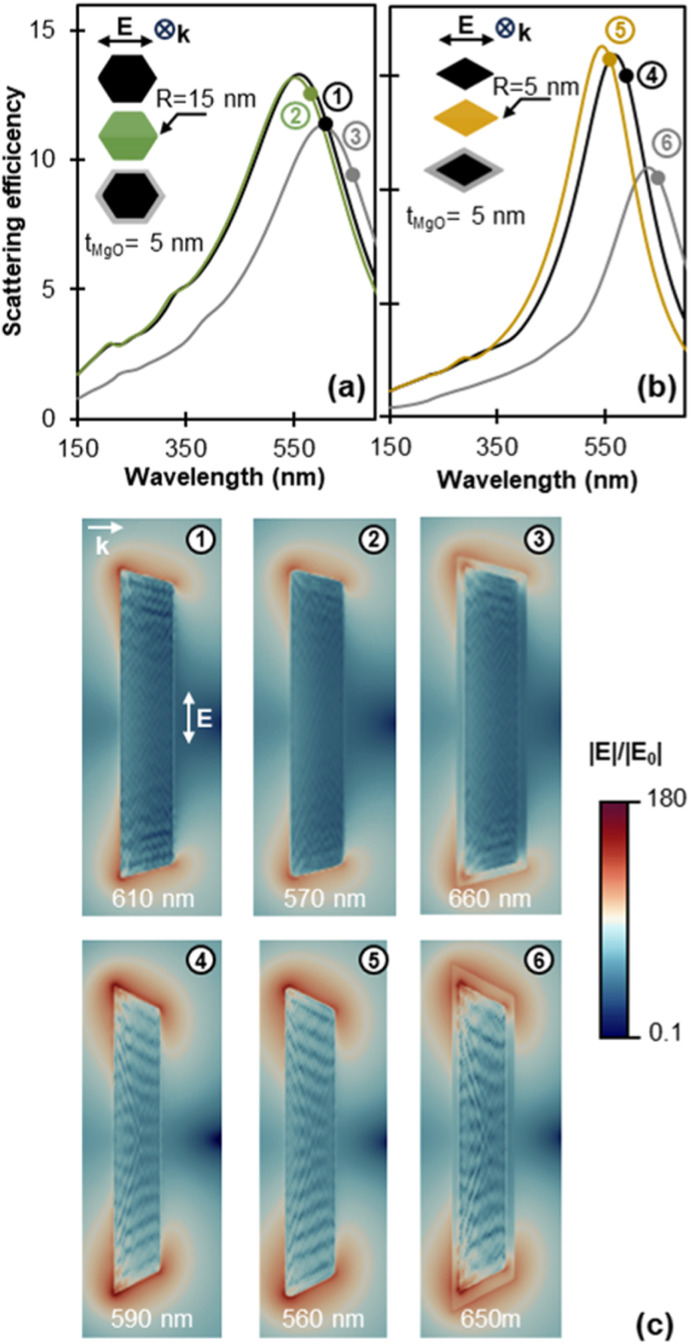
Simulated effects of MgO and tip rounding on the far-field and near-field response. Scattering efficiency for longitudinal polarisation for (a) sharp Mg (black), rounded Mg (green), and sharp Mg@MgO (grey) tent NPs and (b) sharp Mg (black), rounded Mg (yellow), and sharp Mg@MgO (grey) (112̄1) kite NPs. (c) Electric field enhancement for cross sections along the NP twin plane for (1), (4) sharp Mg, (2), (5) rounded Mg, and (3), (6) sharp Mg@MgO tent and (112̄1) kite NPs, respectively. The coloured circles in each scattering spectrum indicate the wavelength position of the maximum field enhancement on the surface of the NP which corresponds to the field maps. Electric field enhancement |*E*|/|*E*_0_| is plotted in logarithmic colour scale. NP length, *L* = 200 nm.

Kite shapes (type II twins) feature four corners, lying on either of the NP's two symmetry planes which exhibit the highest field enhancement for longitudinal (*XY* plane) and transverse (*XZ* plane) polarisation. Kite shapes show a significantly higher electric field enhancement than that of the type I twins, especially for the longitudinal polarisation. For example, for the longitudinal dipole, the (112̄1) kite has a maximum enhancement |*E*|/|*E*_0_|_max_ = 111.2, almost three times the maximum value for the tent. For polarisation along the long axis this is due to kites having sharper tips than the type I twins. Meanwhile, for the transverse polarisation the plasmon now oscillates between corners as opposed to the edge-to-edge oscillations of the type I twins. For longitudinal polarisation, there is significant variation within the kite shapes as well: a 65% (20%) increase in the maximum (average) enhancement occurs between the *y* = 1 and *y* = 4 kites resulting in the largest maximum (average) enhancement of 183.6 (6.33) for the (112̄4) kite. Additionally, for transverse polarisation and similarly to the type I twins, smaller angles trigger a more intense enhancement within the concave angle as a result of strong coupling between the NP corners (Fig. 8g). Previous studies have calculated the maximum field enhancement of Au octahedral NPs (|*E*|/|*E*_0_|_max_ = 40), cubes (30), pentagonal bipyramids (20), rhombic dodecahedra (15), and spheres (6)^58^ as well as for Ag NPs with various shapes, including spheroids (69) and triangles (59).^47^ The maximum enhancement of 184, corresponding to the longitudinal polarisation of the (112̄4) kite, is larger than for the aforementioned shapes, and the same order of magnitude as that calculated for Au nanostars with a tip-to-tip size of ∼100 nm (|*E*|/|*E*_0_|_max_ ∼250).^59^

While the values above are indicative of the potential of kite shapes for field enhancement, the native oxide layer of Mg NPs is expected to significantly affect the enhancement around the NP and must be considered. Fig. 8 shows the far-field and near-field response modification due to a 5 nm oxide layer for a tent and a (112̄1) kite (further details including for the (101̄3) taco and (112̄4) kite can be found in Fig. S16 and S17†). The 5 nm MgO shell was chosen to account for the 3–5 nm MgO thickness reported for gas-phase syntheses,^60,61^ with a thicker oxide layer expected to further redshift the LSPR energy and lower the surface field enhancement. As discussed earlier, the near-field and far-field response do not necessarily peak at the same energy; to enable a straightforward comparison between coated and uncoated NPs we report here the field enhancement at the near-field resonance for both structures (in contrast to Fig. 8 where we considered the far-field resonance). The near-field resonance wavelength is indicated with a coloured disk on the scattering profiles of Fig. 8. A conformal 5 nm MgO layer causes a 40 nm and 60 nm redshift of the (far-field) scattering peak of the tent and kite, respectively. For the near-field enhancement maxima at the NP surface (outside the oxide layer) the redshift due to the oxide is 50 nm and 60 nm for the tent and kite, respectively. Note that the overall maximum field enhancement of MgO-coated NPs, situated within the oxide shell, appears at a slightly different energy than both the maximum surface enhancement and far-field scattering response (Fig. S16†).

The oxide layer also significantly affects the magnitude and distribution of the electric field enhancement around the NP as illustrated in Fig. 8. The maximum enhancement location remains close to the tips of the metal surface while field enhancement decreases through the MgO layer.^62^ When compared to bare structures, shapes with the sharpest tips suffer the most enhancement loss at the surface of the MgO layer (Table S3†). This is attributed to the modelling of the shell as conformal, resulting in higher MgO thickness at the very tip, *i.e.*, the distance between the tip of the core and the tip of the shell is larger than 5 nm. For longitudinal polarisation, the maximum field enhancement on the NP's surface is found to decrease by 67% (from 43.7 to 13.7), 77% (from 81.7 to 18.8), 81% (from 114.4 to 21.5), and 90% (from 183.6 to 18.1) with the addition of an MgO layer for the tent, taco, (112̄1) kite, and (112̄4) kite, respectively. Variations in the average electric field enhancement on the surface of the NPs are not as large; the MgO layer causes a 15% and 40% decrease of the average field enhancement for (112̄1) and (112̄4) kites, respectively, while a modest 4.4% increase and 3.5% decrease are observed for the tent and taco shapes, respectively. The observed increase for the tent can be attributed to field delocalisation around the NP tip of the oxide-coated structures as compared to the bare Mg NPs.

The corner rounding commonly found in synthesised shapes can also affect the predicted enhancement values discussed above. Tip rounding of *R* = 15 nm and *R* = 5 nm were chosen for all the tips of the tent shape and (112̄1) kite shape, respectively. A 3 nm edge rounding was applied as well. The far-field response of the rounded tent shape exhibits a 40 nm blueshift while its maximum near-field enhancement drops from 43.7 to 28.7 (34% drop). For the kite shape, a 30 nm blueshift is observed, accompanied by a drop in the near-field intensity from 114.4 to 92.0 (20% drop). The effects observed in both shapes are comparable despite the rounding radius of the tent being 3 times larger: the kites have much sharper tips in the “perfect” model such that blunting to a fixed rounding diameter has a larger relative effect on the shape, and consequently its optical properties. The scattering efficiency for the kite increases for the *R* = 5 nm tip-rounding applied here (Fig. 8b for (112̄1) kite and Fig. S16† for (112̄4) kite). This is consistent with the increase in scattering intensity previously observed for small tip blunting of Ag triangles and cubes,^63,64^ and related to a narrower linewidth for the rounded shapes (Fig. S17†).

### 3.6. Photothermal effects

**Fig. 9:**
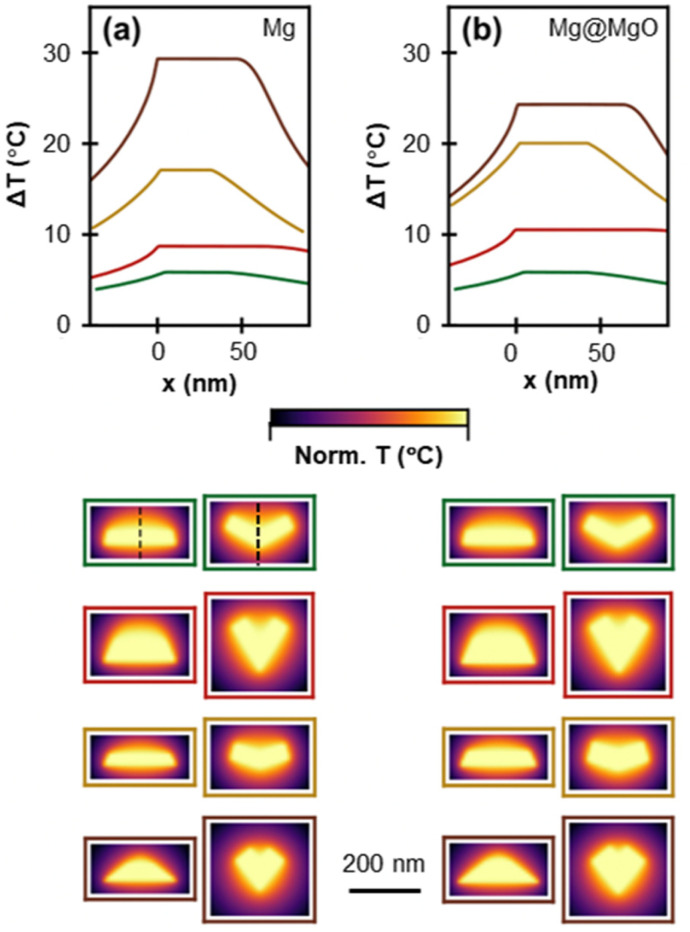
Numerically obtained photothermal response of bare and 5 nm MgO-coated NPs. Temperature profile across the *XY* and *XZ* (shown for the tent shape with dashed line on both cross sections) for (a) bare and (b) MgO-coated (*t* = 5 nm) (101̄1) tent (green), (101̄3) taco (red), (112̄1) kite (yellow), and (112̄4) kite (brown) shapes. Normalised temperature maps on the two symmetry planes of the shapes are shown in colour coded boxes. NP length, *L* = 200 nm.

To unravel the photothermal properties of the studied shapes, we employed t-DDA calculations to investigate the temperature rise (Δ*T*) of (101̄1), (101̄3), (112̄1), and (112̄4) twinned Mg NPs, with and without a 5 nm MgO shell, when irradiated with a 10^7^ W m^−2^ laser at their longitudinal dipole resonance. The temperature maps, normalised for clarity, at the two symmetry planes (*XY* and *XZ* plane, see schematic in Fig. 7) as well as the temperature profile on their intersection are shown in Fig. 9 (non-normalised temperature maps are provided in Fig. S18†). The temperature increases in the order of (101̄1) tent, (101̄3) taco, (112̄1) kite, and (112̄4) kite with corresponding Δ*T* values of 5.9 °C, 8.7 °C, 17.1 °C, and 29.3 °C within the shape (Fig. 9a). This is proportional to the ratio of the absorption cross section to the effective radius of the NP (Fig. S19a†) in accordance to what has been reported in ref.^65^ Additionally, the temperature drop as a function of the distance from the NP surface varies as a function of shape. Higher temperatures are maintained within the cavity formed by the NPs wings, clearly seen from the temperature maps in Fig. 9, especially on the *XY* plane. This effect is stronger for the tent and taco shapes as opposed to the two kites and can be attributed to the shape of the NP wings which have a significantly smaller area for the kites.

The addition of a 5 nm MgO layer results in a change of the plasmon-induced temperature rise with new Δ*T* values of 8.4 °C (42% increase), 10.5 °C (21% increase), 20.1 °C (18% increase) and 24.3 °C (17% decrease) for the (101̄1) tent, (101̄3) taco, (112̄1) kite, and (112̄4) kite, respectively, following again the increase in the ratio of absorption cross section to the NPs’ effective radius (Fig. S19a†). The change in ΔT reflects the slower increase of the absorption cross section of the MgO-coated NPs, as opposed to the bare NPs, as the NP size decreases (Fig. S19b†). Finally, increasing the MgO thickness up to 24 nm results in small, shape-dependent changes in Δ*T* as illustrated in Fig. S20;† for instance, for a 24 nm shell a modest change in the ΔT of 0.5 °C and 3 °C were calculated for a hexagonal prism and (112̄1) kite, respectively.

## 4. Conclusion

To conclude, twinned shapes of Mg, an earth-abundant plasmonic metal, were described using the HCP function of Crystal Creator. The different symmetry of the HCP unit cell as compared to the cubic and tetragonal systems, and the abundance of twin planes in Mg yield an array of unique NP geometries, including concave shapes, strikingly different from those of other plasmonic metals. In particular, we modelled an array of singly twinned Mg shapes formed by twinning along one of the 7 known (in the bulk) Mg twin planes, *i.e.*, the type I pyramidal (101̄*x*), *x* = 1, 2, 3 and type II pyramidal (112̄*y*), *y* = 1, 2, 3, 4 planes. These shapes form concave structures where the folding angle depends on the orientation of the twin plane. Depending on the growth conditions, variants of these shapes are obtained, such as elongated and concave rod-like shapes with variable ARs as well as filled, convex shapes.

The plasmonic behaviour of these twinned shapes was investigated numerically in the far-field and near-field. Shape has a significant effect on the plasmonic modes, and we showed that while concave shapes mostly sustain resonant modes with nodes along their length and/or width, concave shapes with small folding angles and filled shapes also sustain modes with nodes along the NP height. Additionally, the twin plane orientation influences the appearance of the modes by controlling the folding angle and the number of exposed tips. Shape with smaller folding angles exhibit a redshift of their longitudinal plasmon resonance, due to their higher AR, and a strong field localisation in the concave region for transverse polarisation. The studied shapes yield increasingly sharper corners as the twin plane angle with the basal plane decreases, resulting in the strongest maximum field enhancement of 184 observed for a longitudinal polarisation at the tip of a (112̄4) kite. The presence of a 5 nm MgO shell or rounded corners was found to cause a significant shape-dependent decrease of the maximum field enhancement on the NP's surface. On the contrary, the presence of a MgO shell can have a positive effect on the photothermal response of Mg NPs and can increase the plasmon-induced temperature rise by up to 42% among the studied shapes, as compared to bare Mg NPs. Overall, these results are instructive for the design and potential use of Mg NPs in enhanced spectroscopies and photothermal applications.

## Conflicts of interest

There are no conflicts to declare.

## Supplementary Material

NR-016-D3NR05848D-s001

## References

[cit1] Link S., El-Sayed M. A. (1999). J. Phys. Chem. B.

[cit2] Brioude A., Jiang X. C., Pileni M. P. (2005). J. Phys. Chem. B.

[cit3] Nicoletti O., De La Peña F., Leary R. K., Holland D. J., Ducati C., Midgley P. A. (2013). Nature.

[cit4] Cobley C. M., Au L., Chen J., Xia Y. (2010). Expert Opin. Drug Delivery.

[cit5] Mayer K. M., Hafner J. H. (2011). Chem. Rev..

[cit6] Swearer D. F., Zhao H., Zhou L., Zhang C., Robatjazi H., Martirez J. M. P., Krauter C. M., Yazdi S., McClain M. J., Ringe E., Carter E. A., Nordlander P., Halas N. J. (2016). Proc. Natl. Acad. Sci. U. S. A..

[cit7] Sharma B., Frontiera R. R., Henry A.-I., Ringe E., Van Duyne R. P. (2012). Mater. Today.

[cit8] Blaber M. G., Arnold M. D., Ford M. J. (2010). J. Phys.: Condens. Matter.

[cit9] Ringe E. (2020). J. Phys. Chem. C.

[cit10] Hopper E. R., Boukouvala C., Asselin J., Biggins J. S., Ringe E. (2022). J. Phys. Chem. C.

[cit11] Sterl F., Strohfeldt N., Walter R., Griessen R., Tittl A., Giessen H. (2015). Nano Lett..

[cit12] Duan X., Liu N. (2019). Acc. Chem. Res..

[cit13] Locatelli E., Matteini P., Sasdelli F., Pucci A., Chiariello M., Molinari V., Pini R., Franchini M. C. (2014). Chem. Commun..

[cit14] Biggins J. S., Yazdi S., Ringe E. (2018). Nano Lett..

[cit15] Ringe E., Langille M. R., Sohn K., Zhang J., Huang J., Mirkin C. A., Van Duyne R. P., Marks L. D. (2012). J. Phys. Chem. Lett..

[cit16] Seo D., Il Yoo C., Chung I. S., Park S. M., Ryu S., Song H. (2008). J. Phys. Chem. C.

[cit17] Mott D., Galkowski J., Wang L., Luo J., Zhong C. J. (2007). Langmuir.

[cit18] González A. L., Noguez C. (2007). Phys. Status Solidi C.

[cit19] Lu S., Yu H., Gottheim S., Gao H., Desantis C. J., Clark B. D., Yang J., Jacobson C. R., Lu Z., Nordlander P., Halas N. J., Liu K. (2018). J. Am. Chem. Soc..

[cit20] McClain M. J., Schlather A. E., Ringe E., King N. S., Liu L., Manjavacas A., Knight M. W., Kumar I., Whitmire K. H., Everitt H. O., Nordlander P., Halas N. J. (2015). Nano Lett..

[cit21] Asselin J., Boukouvala C., Hopper E. R., Ramasse Q. M., Biggins J. S., Ringe E. (2020). ACS Nano.

[cit22] Wulff G. (1901). Z. Kristallogr..

[cit23] Sekerka R. F. (2005). Cryst. Res. Technol..

[cit24] Marks L. D. (1983). J. Cryst. Growth.

[cit25] Ringe E., Van Duyne R. P., Marks L. D. (2013). J. Phys. Chem. C.

[cit26] Boukouvala C., Daniel J., Ringe E. (2021). Nano Converg..

[cit27] Liu W., Aguey-Zinsou K. F. (2014). J. Mater. Chem. A.

[cit28] Norberg N. S., Arthur T. S., Fredrick S. J., Prieto A. L. (2011). J. Am. Chem. Soc..

[cit29] Yang N., Gong F., Liu B., Hao Y., Chao Y., Lei H., Yang X., Gong Y., Wang X., Liu Z., Cheng L. (2022). Nat. Commun..

[cit30] Ohno T., Yamauchi K. (1981). Jpn. J. Appl. Phys..

[cit31] Barbillon G., Ivanov A., Sarychev A. K. (2020). Symmetry.

[cit32] Blanchard R., Aoust G., Genevet P., Yu N., Kats M. A., Gaburro Z., Capasso F. (2012). Phys. Rev. B: Condens. Matter Mater. Phys..

[cit33] Rashidi A., Chryssomallis M. T., Anagnostou D. E. (2014). J. Opt. Soc. Am. A.

[cit34] Sung J., Hicks E. M., Van Duyne R. P., Spears K. (2008). J. Phys. Chem. C.

[cit35] Husu H., Mäkitalo J., Laukkanen J., Kuittinen M., Kauranen M. (2010). Opt. Express.

[cit36] Lévesque Q., Makhsiyan M., Bouchon P., Pardo F., Jaeck J., Bardou N., Dupuis C., Haïdar R., Pelouard J. L. (2014). Appl. Phys. Lett..

[cit37] Wiecha P. R., Black L. J., Wang Y., Paillard V., Girard C., Muskens O. L., Arbouet A. (2017). Sci. Rep..

[cit38] Sukharev M., Sung J., Spears K. G., Seideman T. (2007). Phys. Rev. B: Condens. Matter Mater. Phys..

[cit39] Black L. J., Wiecha P. R., Wang Y., De Groot C. H., Paillard V., Girard C., Muskens O. L., Arbouet A. (2015). ACS Photonics.

[cit40] Partridge P. G. (1967). Metall. Rev..

[cit41] Christian J. W. , in The Theory of Transformations in Metals and Alloys, Elsevier Science, Oxford, 2nd edn, 1981, pp. 859–960

[cit42] Boukouvala C. , Hopper E. R. and Ringe E., Crystal Creator, Optical Nanomaterials Group, Cambridge, UK, https://www.on.msm.cam.ac.uk/code.html

[cit43] Draine B. T., Flatau P. J. (2008). J. Opt. Soc. Am. A.

[cit44] Bigelow N. W., Vaschillo A., Iberi V., Camden J. P., Masiello D. J. (2012). ACS Nano.

[cit45] Baldwin C. L., Bigelow N. W., Masiello D. J. (2014). J. Phys. Chem. Lett..

[cit46] Palik E. D. , Handbook of optical constants of solids, Academic Press, 1998

[cit47] Hao E., Schatz G. C. (2004). J. Chem. Phys..

[cit48] Hopper E. R., Wayman T. M. R., Asselin J., Pinho B., Boukouvala C., Torrente-Murciano L., Ringe E. (2022). J. Phys. Chem. C.

[cit49] de la Peña F., Prestat E., Fauske V. T., Burdet P., Lähnemann J., Jokubauskas P., Furnival T., Nord M., Ostasevicius T., MacArthur K. E., Johnstone D. N., Sarahan M., Aarholt T., Taillon J., pquinn-dls V., Eljarrat A., Caron J., Francis C., Nemoto T., Poon T., Mazzucco S., actions-user N., Cautaerts N., Somnath S., Slater T., Walls M. (v1.7.1). Winkler and DENSmerijn, hyperspy/hyperspy: Release.

[cit50] Lautar A. K., Kopač D., Rejec T., Bančič T., Dominko R. (2019). Phys. Chem. Chem. Phys..

[cit51] Boukouvala C., Ringe E. (2019). J. Phys. Chem. C.

[cit52] Ghildiyal P., Biswas P., Herrera S., Xu F., Alibay Z., Wang Y., Wang H., Abbaschian R., Zachariah M. R. (2022). ACS Appl. Mater. Interfaces.

[cit53] West C. A., Olafsson A., Pakeltis G., Garfinkel D. A., Rack P. D., Masiello D. J., Camden J. P., Idrobo J. C. (2020). J. Phys. Chem. C.

[cit54] Liu F., Zhang X., Fang X. (2017). Sci. Rep..

[cit55] Zheng Y. B., Juluri B. K., Mao X., Walker T. R., Huang T. J. (2008). J. Appl. Phys..

[cit56] Shokova M. A., Bochenkov V. E. (2021). Nanomaterials.

[cit57] Zuloaga J., Nordlander P. (2011). Nano Lett..

[cit58] Montaño-Priede J. L., Pal U. (2019). J. Phys. Chem. C.

[cit59] Hao F., Nehl C. L., Hafner J. H., Nordlander P. (2007). Nano Lett..

[cit60] Kooi B. J., Palasantzas G., De Hosson J. T. M. (2006). Appl. Phys. Lett..

[cit61] Venturi F., Calizzi M., Bals S., Perkisas T., Pasquini L. (2015). Mater. Res. Express.

[cit62] Gutierrez Y., Ortiz D., Sanz J. M., Saiz J. M., Gonzalez F., Everitt H. O., Moreno F. (2016). Opt. Express.

[cit63] Grześkiewicz B., Ptaszyński K., Kotkowiak M. (2014). Plasmonics.

[cit64] Geddes C. D., Lakowicz J. R. (2002). J. Fluoresc..

[cit65] Baffou G., Quidant R., García De Abajo F. J. (2010). ACS Nano.

