# Supplementary material for: Far-field, near-field and photothermal response of plasmonic twinned magnesium nanostructures
Source: Nanoscale. 2024 Jan 31;16(15):7480–92. doi: 10.1039/d3nr05848d (PMC11025716; doi:10.1039/d3nr05848d)
Supplement: NR-016-D3NR05848D-s001 [file NR-016-D3NR05848D-s001.pdf]

Electronic Supplementary Material (ESI) for Nanoscale.
This journal is © The Royal Society of Chemistry 2024

Electronic Supplementary Information for

Far-Field, Near-Field and Photothermal Response of Plasmonic Twinned Magnesium Nanostructures

Christina Boukouvala,[a] Claire A. West,[a] Andrey Ten,[a] Elizabeth R. Hopper,[a,b] Quentin M. Ramasse,[c] John S. Biggins,[d] and Emilie Ringe[a*]

[a]Department of Materials Science and Metallurgy, University of Cambridge, 27 Charles Babbage Road, Cambridge, CB3 0FS and Department of Earth Sciences, Downing Street, Cambridge, CB2 3EQ, United Kingdom

[b]Department of Chemical Engineering and Biotechnology, University of Cambridge, Philippa Fawcett Drive, Cambridge, United Kingdom, CB3 0AS

[c]School of Chemical and Process Engineering, University of Leeds, 211 Clarendon Road, Leeds, United Kingdom, LS2 9JT, School of Physics and Astronomy, University of Leeds, Woodhouse, Leeds, United Kingdom, LS2 9JS and SuperSTEM, SciTech Daresbury Science and Innovation Campus, Keckwick Lane, Warrington, United Kingdom, WA4 4AD.

[d]Department of Engineering, University of Cambridge, Trumpington Street, Cambridge, CB2 1PZ, United Kingdom

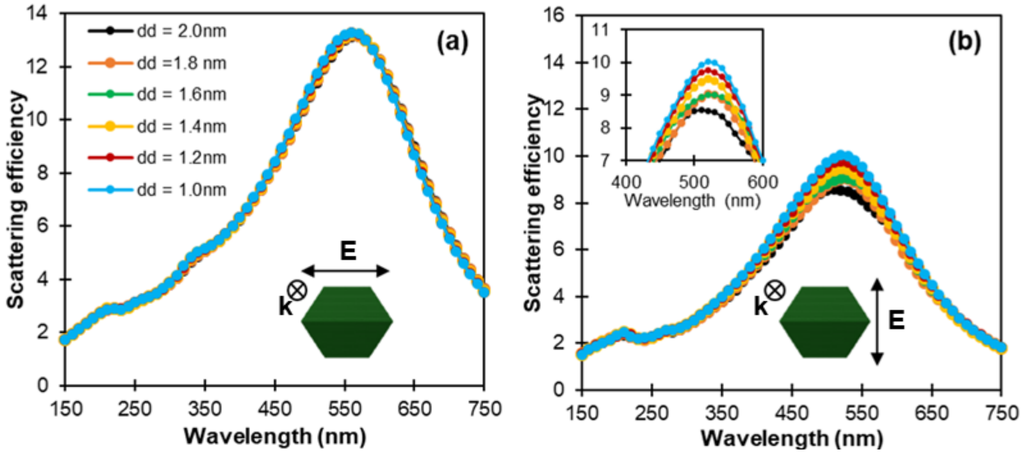

Figure S1. Convergence of scattering efficiency as a function of interdipole distance dd. Scattering spectra for a 200 nm long tent shape excited with (a) longitudinal and (b) transverse polarization as shown in the insets. Out of plane light propagation direction is indicated with ⊗. NP length, L = 200 nm.

Table S1. Shapes, dimensions, DDA parameters and Wulff parameters for the NPs used in Figures 2-6. L: Tip-to-tip length, AR: aspect ratio, t: thickness, aeff: Effective radius (DDA input), f: filled, and 1, 2, 3 correspond to the progressively filled chair shapes.

Shape	$\frac{a_{0001}}{a_{10\bar{1}0}}$	re-entrant	twin	L (nm)	AR	t (nm)	H (nm)	a_{eff} (nm)
$(10\bar{1}1)$	4.87/30	0.06	0	200	1.27	28	70	55.9
$(10\bar{1}1)$f	4.87/30	10	0	200	1.27	-	190	78.7
$(10\bar{1}2)$	4.72/30	0.15	0	200	1.58	28	89	53.2
$(10\bar{1}2)$-1	4.72/30	1	0	200	1.58	41	98	57.9
$(10\bar{1}2)$-2	4.72/30	2	0	200	1.58	54	107	60.9
$(10\bar{1}2)$-3	4.72/30	3	0	200	1.58	67	116	62.6
$(10\bar{1}2)$f	4.72/30	10	0	200	1.58	-	129	63.4
$(10\bar{1}3)$	4.69/30	0.23	0	200	1.95	28	99	51.7
$(11\bar{2}1)$	4/30	0.15	1.13	200	1.78	28	42	35.6
$(11\bar{2}1)$f	4/30	10	1.13	200	1.78	-	213	57.9
$(11\bar{2}2)$	4/30	0	1.24	200	1.99	28	51	38.0
$(11\bar{2}3)$	3.5/30	1	1.4	200	2.36	28	61	35.5
$(11\bar{2}4)$	3.5/30	1.2	1.6	200	2.70	28	67	33.4

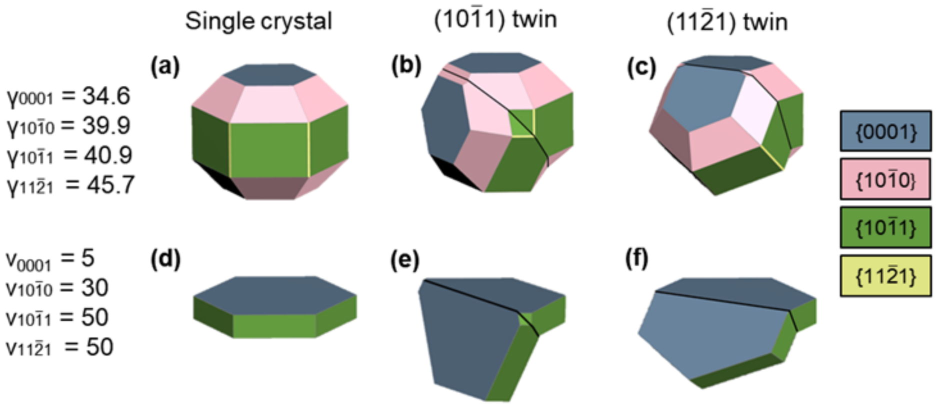

Figure S2. Mg nanoparticle twinning in the thermodynamic and kinetic Wulff construction. Mg shapes in the (a) thermodynamic Wulff construction, based on surface energy values from ref.[1] (b) and (c) modified thermodynamic Wulff construction for a $(10\bar{1}1)$ and a $(11\bar{2}1)$ twin plane, respectively, (d) kinetic Wulff construction with growth velocities based on observed shapes, and (e)-(f) modified kinetic Wulff construction for a $(10\bar{1}1)$ and a $(11\bar{2}1)$ twin plane, respectively. Shapes created in WinXMorph.[2]

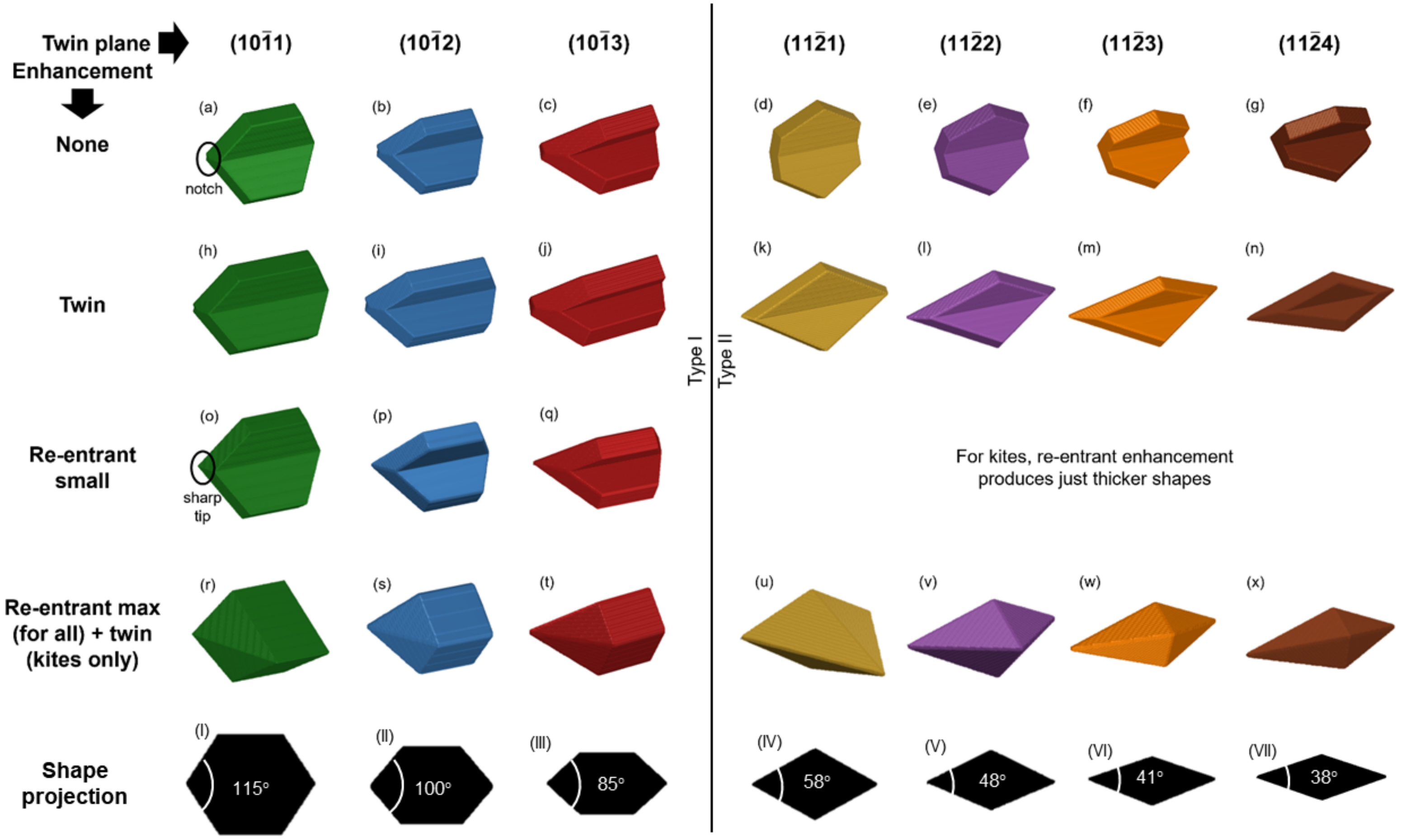

Figure S3. Models of twinned Mg nanoparticle shapes for the seven twin planes, colour coded based on the twin planes in Figure 1b, and with various combinations of re-entrant and twin enhancements as indicated. The last row shows the projection and projected tip angle of the shapes on a plane perpendicular to the twin plane.

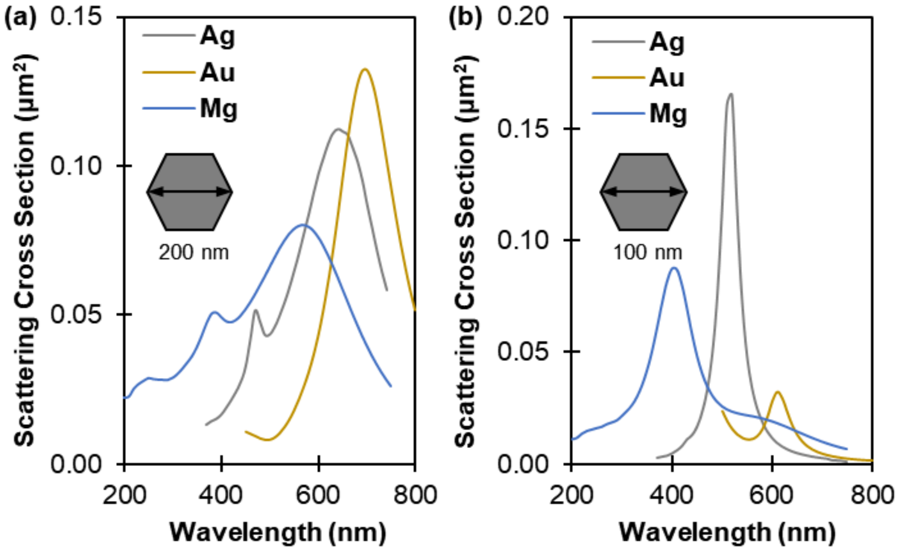

Figure S4. Calculated scattering cross sections for three averaged orthogonal light propagation directions, with two orthogonal polarizations each, for Ag (grey), Au (yellow), and Mg (blue) hexagonal prisms with tip-to-tip length of (a) 200 nm and (b) 100 nm.

Detailed mode description for a tent NP: For a tent NP, the lowest energy peak at 2.1 eV corresponds to (1,0,0), a longitudinal dipole, strongly excited either by light polarized along the long axis or an electron travelling near a tip (Figure 2a, S5a). The second lowest energy mode at 2.3 eV is (0,1,0), a transverse dipole, excited either by light polarized along the short axis or an electron travelling near the long edge. A node across the vertical axis of the NP appears at 3.2 eV for a vertical polarization direction; this is the weak (0,0,1) mode, termed the first symmetric (or antibonding) mode for flat V-shaped structures as opposed to the (0,1,0) being often called the antisymmetric (or bonding) mode.[32,33,39] Next are the (2,0,0) and (1,1,0) modes; these are very close in energy at 3.2 and 3.3 eV, however they are distinguished by their different excitation pattern: the (1,1,0) appears with the electron beam at a corner while the (2,0,0) is strongly excited by a beam at the centre of the long edge. The (1,1,0) can also be strongly excited by light with a transverse polarization and travelling along the long NP axis. The next two modes can be predominantly excited by an electron beam, one mode at the tip (3.6 eV) and another at the edge (4.7 eV) of the tent shape. Additional views of the mode symmetry (Figure S5c) suggest their classification as (3,0,0) and (2,1,0) modes. Finally, at 4.7 eV, a breathing mode, *i.e.,* a mode with radial symmetry for which the (l,w,h) notation is poorly suited, can be excited when the electron beam is travelling through the centre of the NP. Note that while e-DDA does not take into account changes in the propagation of the beam within the NP,[43] it nevertheless produces an interpretable and realistic mode symmetry and energy.

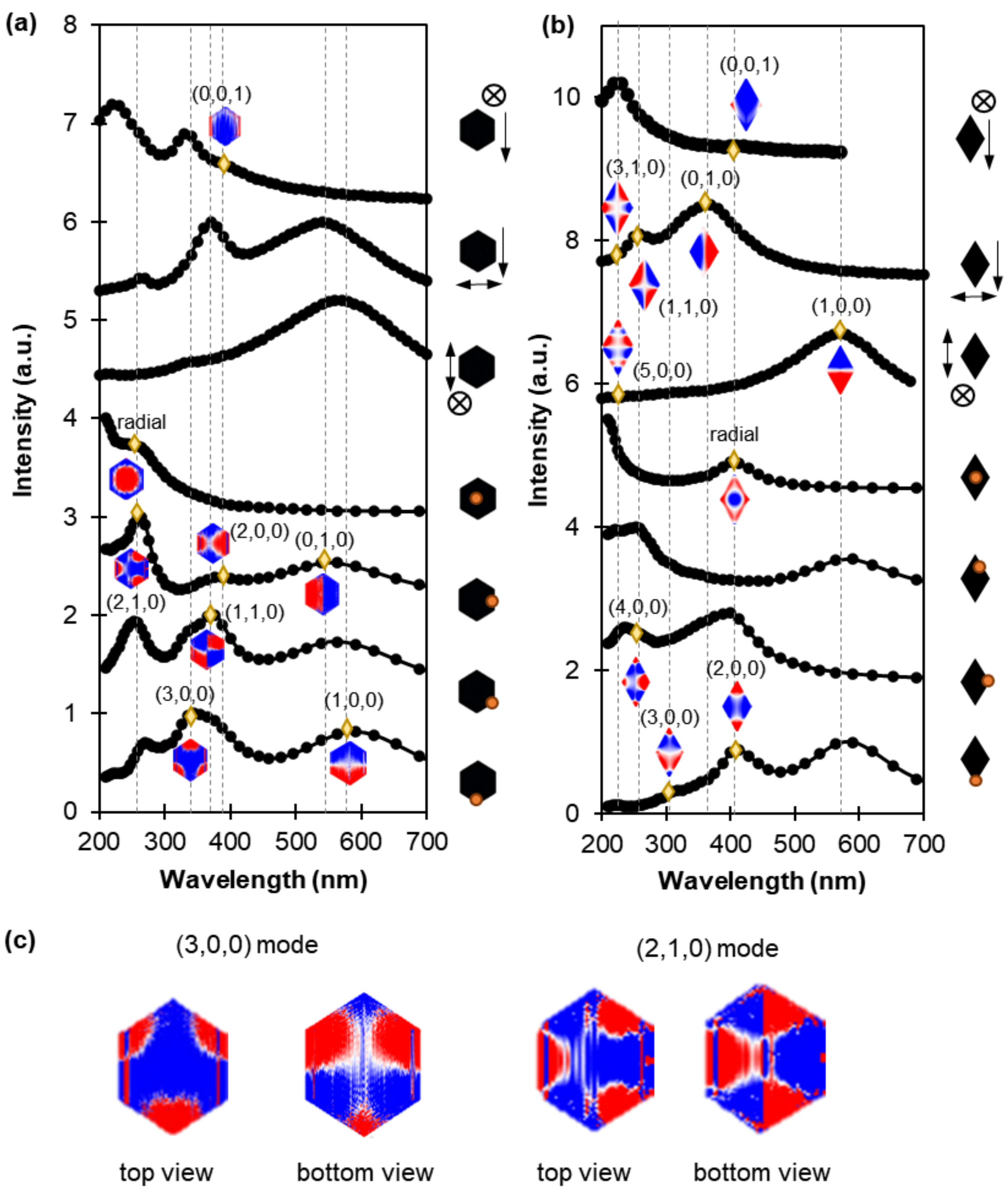

Figure S5. Plasmonic response of the L = 200 nm tent (a) and ($11\bar{2}1$) kite (b) shapes. On top, calculated extinction spectra obtained from an optical excitation for various light propagation directions (single arrow) and polarization directions (double arrow). Yellow diamonds indicate the position where each mode was calculated. Out of plane light propagation and polarization directions are indicated with a ⊗. At the bottom, EEL spectra obtained from an electron beam excitation for various beam positions shown with an orange dot. (c) Top and bottom view of the (3,0,0) and (2,1,0) modes for the tent shape.

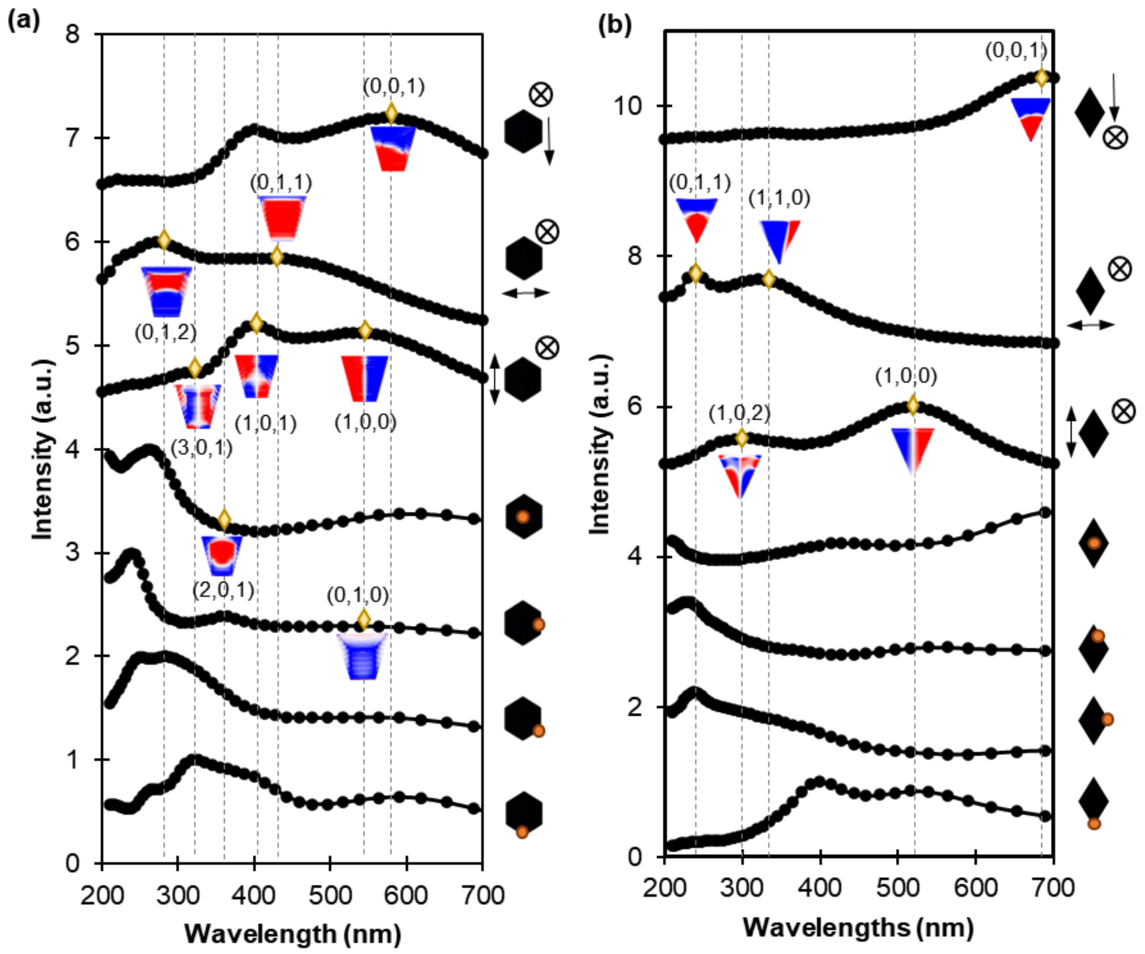

Figure S6. Plasmonic response of the L =200 nm filled tent (a) and filled $(11\bar{2}1)$ kite (b) shapes. On top, calculated scattering spectra obtained from an optical excitation for various light propagation directions (single arrow) and polarization directions (double arrow). Yellow diamonds indicate the position where each mode was calculated. Out of plane light propagation and polarization directions are indicated with a ⊗. At the bottom, EEL spectra obtained from an electron beam excitation for various beam positions shown with an orange dot.

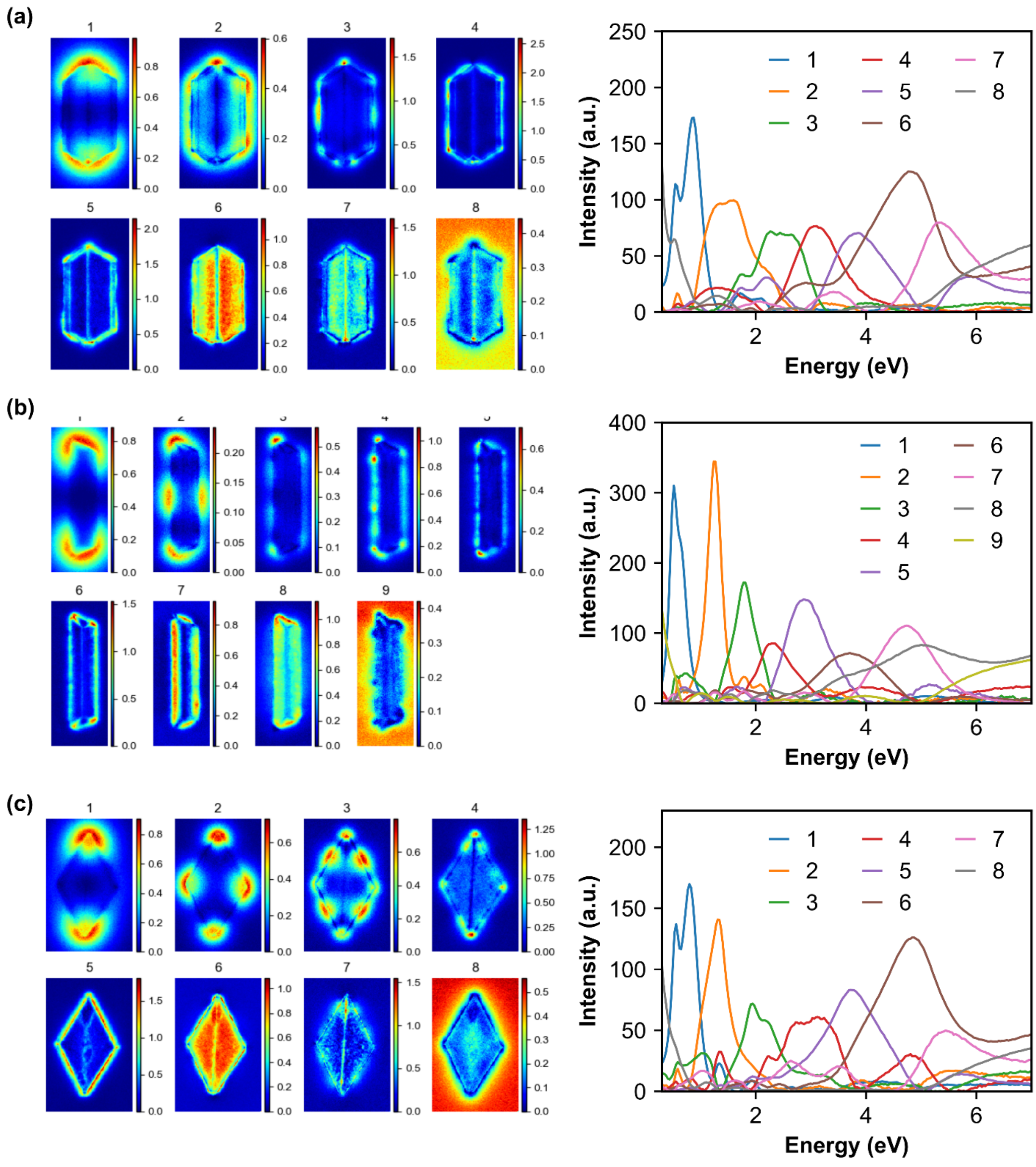

Figure S7 Low-loss STEM-EELS NMF spatial loadings and their spectral components for (a) L = 475 nm tent, (b) L = 792 nm taco, and (c) L = 643 nm ($11\bar{2}1$) kite Mg NPs.

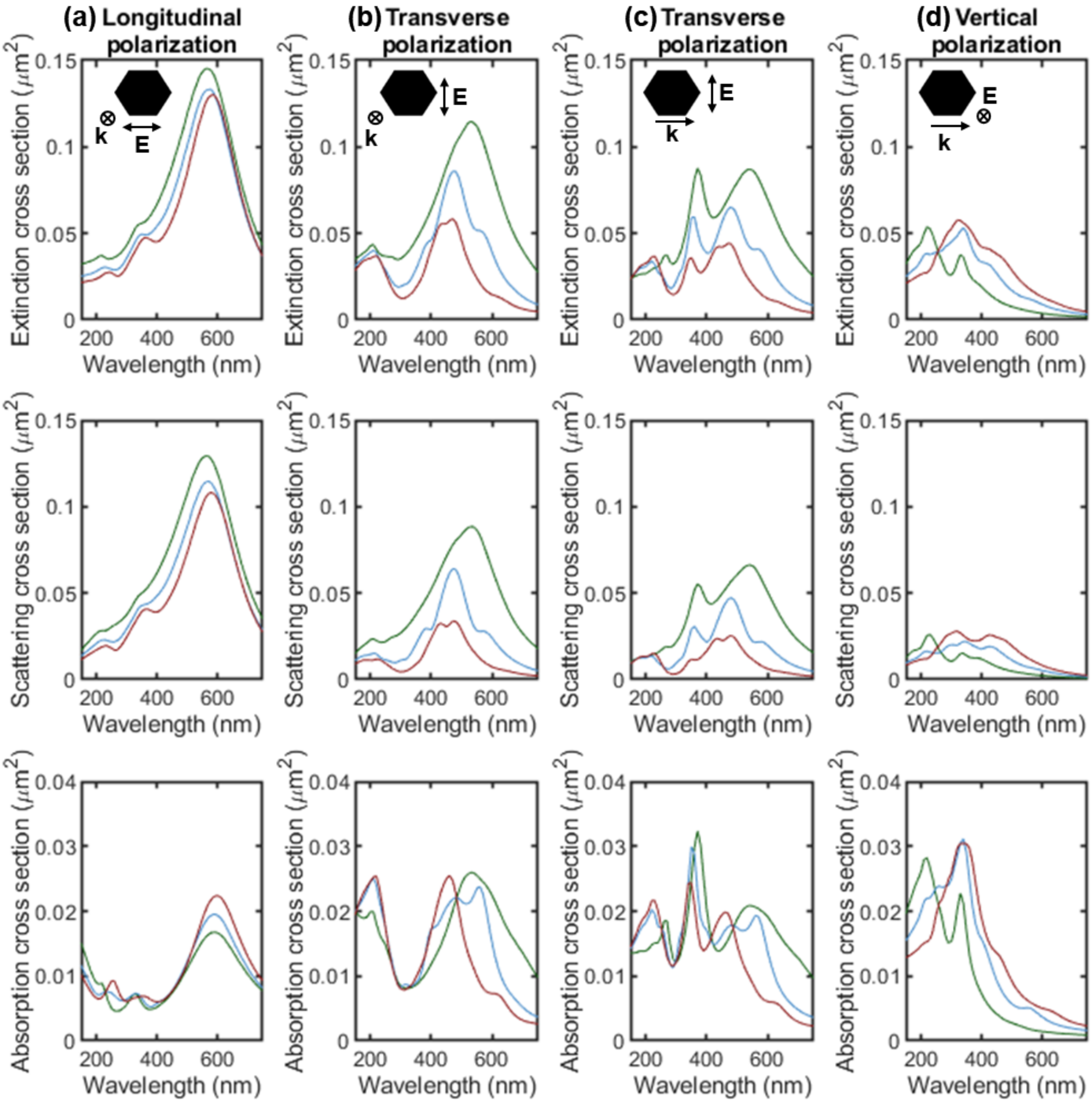

Figure S8. Extinction, scattering and absorption cross sections for a L = 200 nm tent (green), chair (blue), and taco (red) shape and different polarization and for light propagation directions as indicated in the insets. (a) Longitudinal polarization with light propagating along the vertical axis, (b) transverse polarization with light propagating along the vertical axis, (c) transverse polarization with light propagating along the longitudinal axis, and (d) vertical polarization with light propagating along the longitudinal axis.

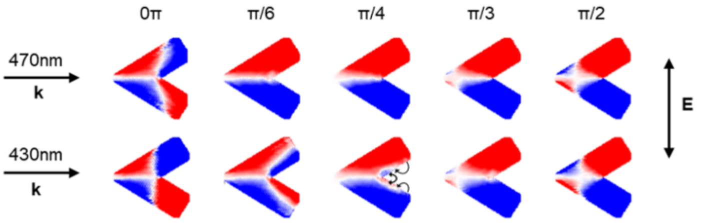

Figure S9. Charge distribution for different phases of the light field for the transverse modes of a Mg taco shape which appear for the (0,1,1) mode at 470 nm (2.6 eV, top), and the tri-dipole mode 430 nm (2.9 eV, bottom). Single and double straight arrows indicate the light propagation and polarization directions, respectively. The curved arrows at phase π/4 indicate the dipole interaction within the "nanocavity" for the tri-dipole mode.

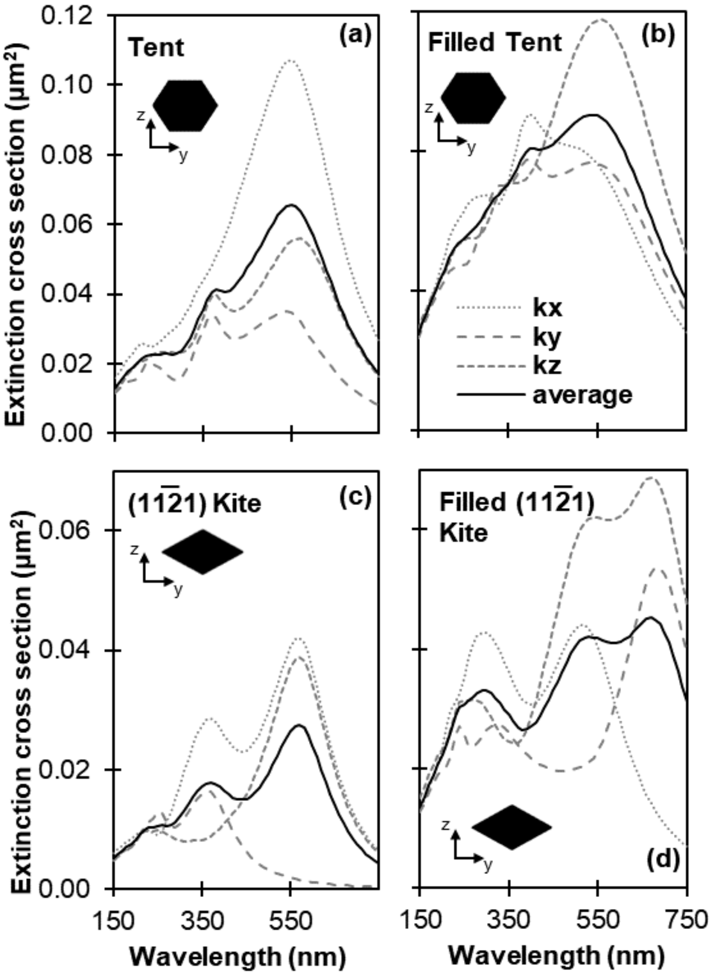

Figure S10. Calculated extinction cross sections for three orthogonal propagation directions $\mathbf{k}_x$, $\mathbf{k}_y$, $\mathbf{k}_z$ with two orthogonal polarizations each (dashed lines), and their average (solid line) for a L = 200 nm (a) tent (b) (11$\bar{2}$1) kite (c) filled tent, and (d) filled (11$\bar{2}$1) kite.

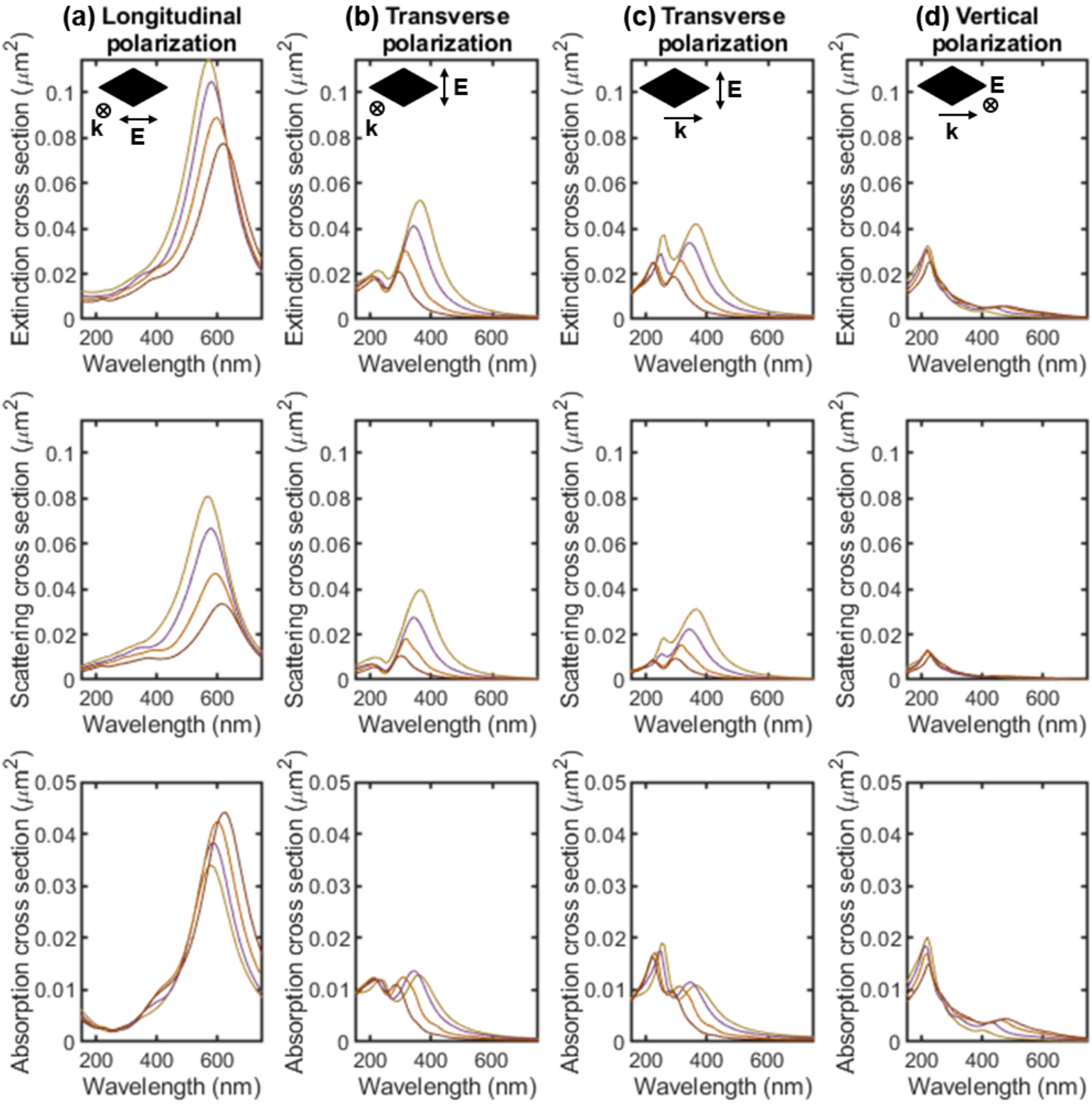

Figure S11. Extinction, scattering and absorption cross sections for a L = 200 nm (11$\bar{2}$1) (yellow), (11$\bar{2}$2) (purple), (11$\bar{2}$3) (orange) and (11$\bar{2}$4) (brown) kite shape and for different polarization and light propagation directions as indicated in the insets. (a) Longitudinal polarization with light propagating along the vertical axis, (b) transverse polarization with light

propagating along the vertical axis, (c) transverse polarization with light propagating along the longitudinal axis, and (d) vertical polarization with light propagating along the longitudinal axis.

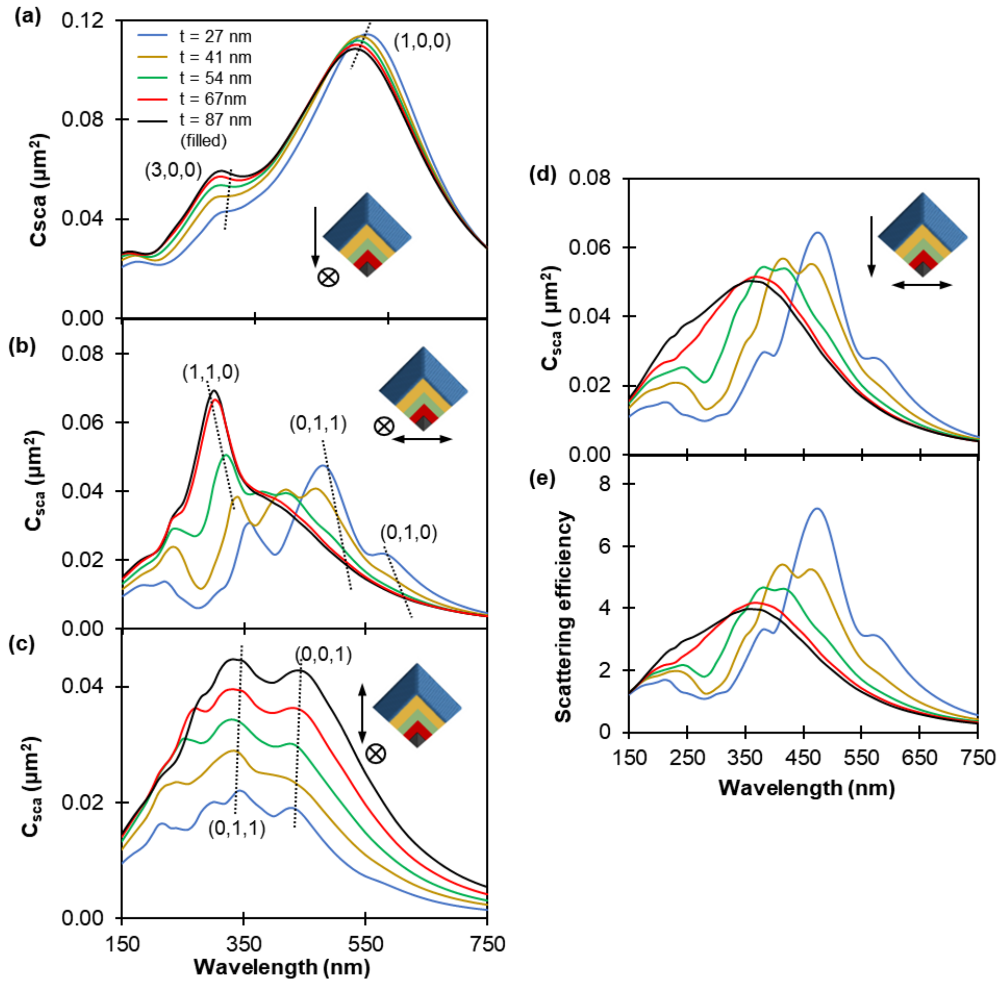

Figure S12. Calculated scattering cross sections and efficiencies obtained from an optical excitation for progressively filled chair shaped particles (L = 200 nm, W = 127 nm). (a) Longitudinal polarization and vertical light propagation direction, (b) transverse polarization and longitudinal light propagation direction, (c) vertical polarization and longitudinal light propagation direction, and (d) and (e) transverse polarization with vertical light propagation direction. Insets illustrate the NPs' cross section overlaid on top of each other such that their thickness increases in the order of blue (thinnest chair), yellow, green, red, and black (filled chair). Spectra are colour coded accordingly and the legend reports the total NP thickness.

Polarization directions and light propagation directions are indicated with double and single arrow, respectively and with ⊗ when out of plane.

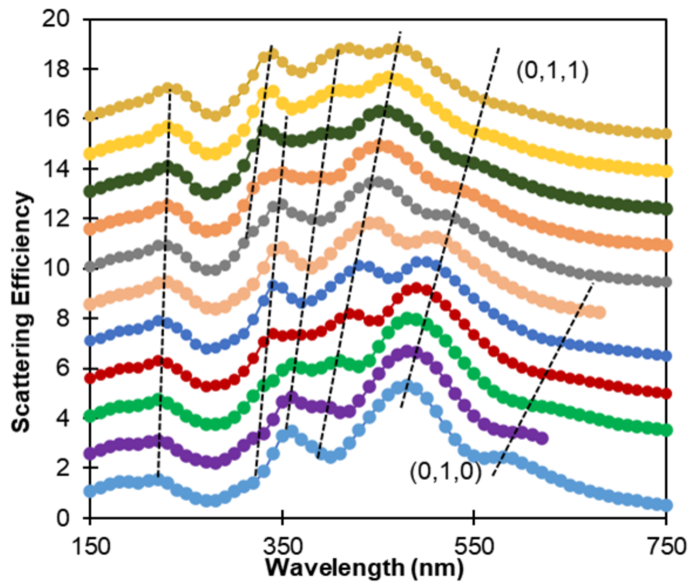

Figure S13. Calculated scattering efficiency for a chair shaped particle (L = 200 nm, W = 127 nm) with increasing thickness from bottom (t = 28 nm) to top (t = 41 nm) obtained from an optical excitation along the long NP axis and transverse polarization direction.

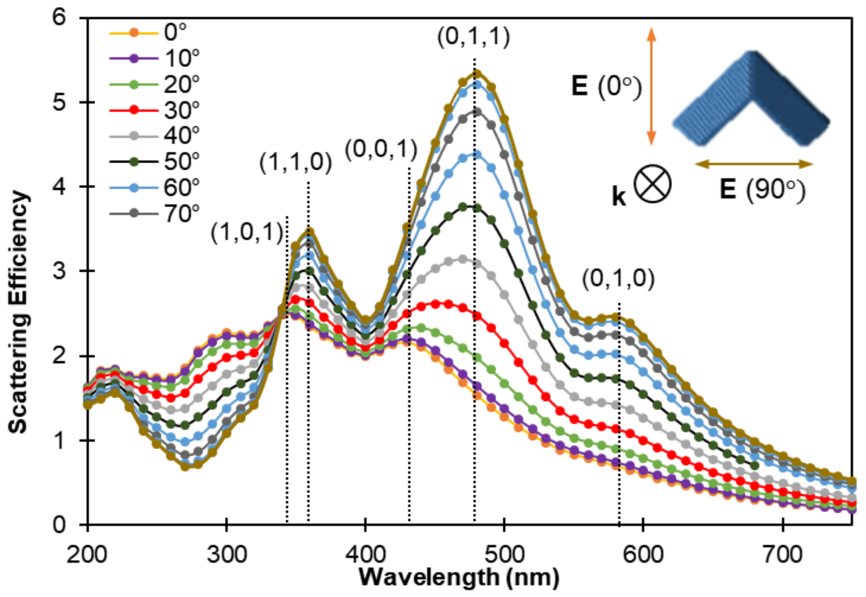

Figure S14. Calculated scattering efficiency for a chair shaped particle (L = 200 nm, W = 127 nm) obtained for an optical excitation along the long NP axis and polarization direction of different angles as indicated by the double arrows.

Table S2. Maximum and average field enhancement values ($|E|/|E_0|$) for type I and type II twins. Dark and light grey rows correspond to longitudinal and transverse polarization, respectively.

	LSPR (nm)	$\vert E\vert/\vert E_0\vert_{max}$	$\vert E\vert/\vert E_0\vert_{average\ surf}$
**tent**	560	39.9	3.03
	520	25.6	3.08
**chair**	570	63.0	3.35
	470	27.4	3.00
**taco**	580	79.4	3.62
	440	27.9	3.18
**($11\bar{2}1$) - kite**	570	111.2	5.29
	360	47.2	3.01
**($11\bar{2}2$)- kite**	580	115.2	5.87
	340	35.6	3.00
**($11\bar{2}3$) - kite**	600	165.3	6.00
	320	33.7	2.92
**($11\bar{2}4$) - kite**	630	183.6	6.33
	290	40.1	2.89

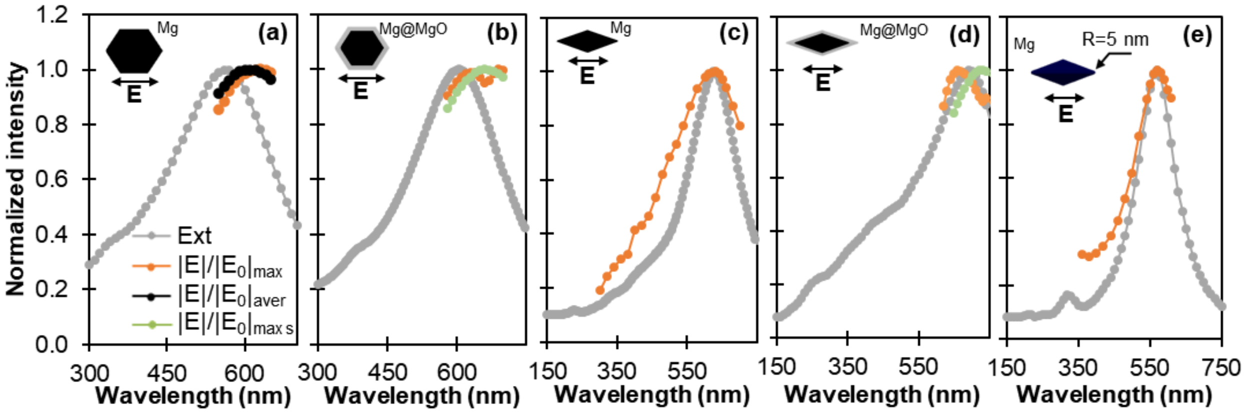

Figure S15 Comparison of the wavelength dependence of the far-field and near-field plasmonic response. Scattering (grey), maximum near-field enhancement (orange), average near-field enhancement on the NP surface (black, shown only for (a)) and maximum near-field enhancement on the NP surface (green) for a (a) Mg tent, (b) Mg tent with 5 nm MgO shell,

(c) (11$\bar{2}$4) kite, and (d) (11$\bar{2}$4) kite with 5 nm MgO shell, polarized along their long axis. The normalized maximum enhancement and maximum enhancement on NP's surface for (a), (b), (c), and (e) are identical. NP (core) length, L = 200 nm.

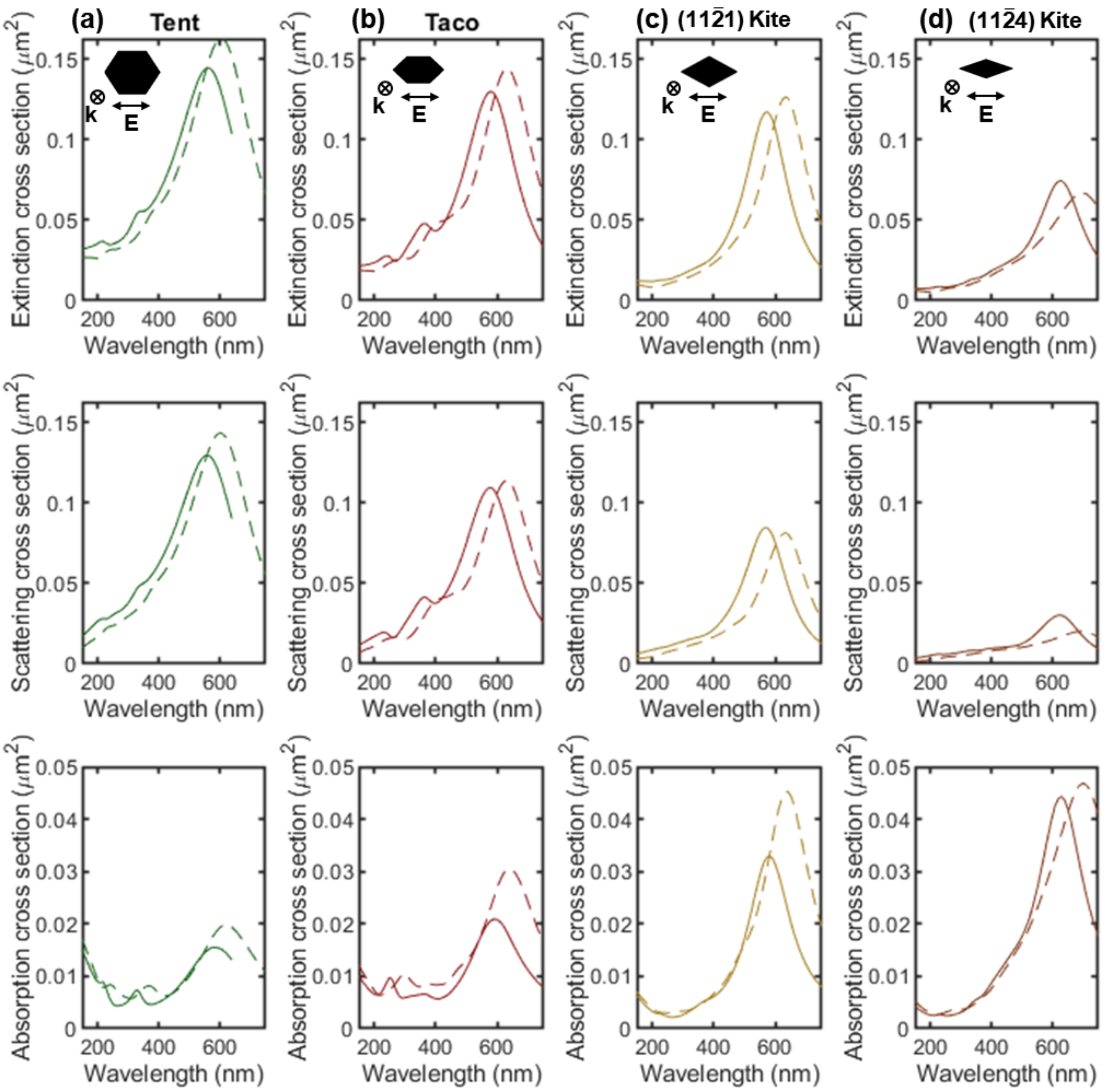

Figure S16. Extinction, scattering and absorption cross sections for longitudinal polarization for a L = 200 nm (a) tent, (b) taco, (c) (11$\bar{2}$1) kite, and (d) (11$\bar{2}$4) kite without (solid) and with (dashed) a 5 nm MgO layer.

Table S3. Maximum and average field enhancement values (|E|/|E$_0$|) for tent, taco, ($11\bar{2}1$) kite, and ($11\bar{2}4$) kite with and without a conformal 5 nm MgO layer and with rounded tips. "Surf" indicates the average and maximum enhancement on the MgO surface. |E|/|E$_0$| max is reported at the wavelength of maximum surface enhancement.

	Peak (nm)	$\lvert E\rvert/\lvert E_0\rvert_{max}$	$\lvert E\rvert/\lvert E_0\rvert_{av\ surf}$	$\lvert E\rvert/\lvert E_0\rvert_{max\ surf}$
**tent**	610	43.7	3.15	43.7
tent rounded (r=15nm)	570	28.7	3.16	28.7
tent MgO (t=5nm)	660	27.5	3.29	13.7
**taco**	600	81.7	3.72	81.7
taco rounded (r=5nm)	590	40.6	3.63	40.6
taco MgO (t=5nm)	670	89.4	3.59	18.8
**($11\bar{2}1$) - kite**	590	114.4	5.41	114.4
($11\bar{2}1$) rounded (r=5nm)	560	63.5	5.36	63.5
($11\bar{2}1$) MgO (t=5nm)	650	95.0	4.58	21.5
**($11\bar{2}4$) - kite**	630	183.6	6.33	183.6
($11\bar{2}4$) rounded (r=5nm)	570	90.2	6.93	90.2
($11\bar{2}4$) MgO (t=5nm)	720	95.9	3.81	18.1

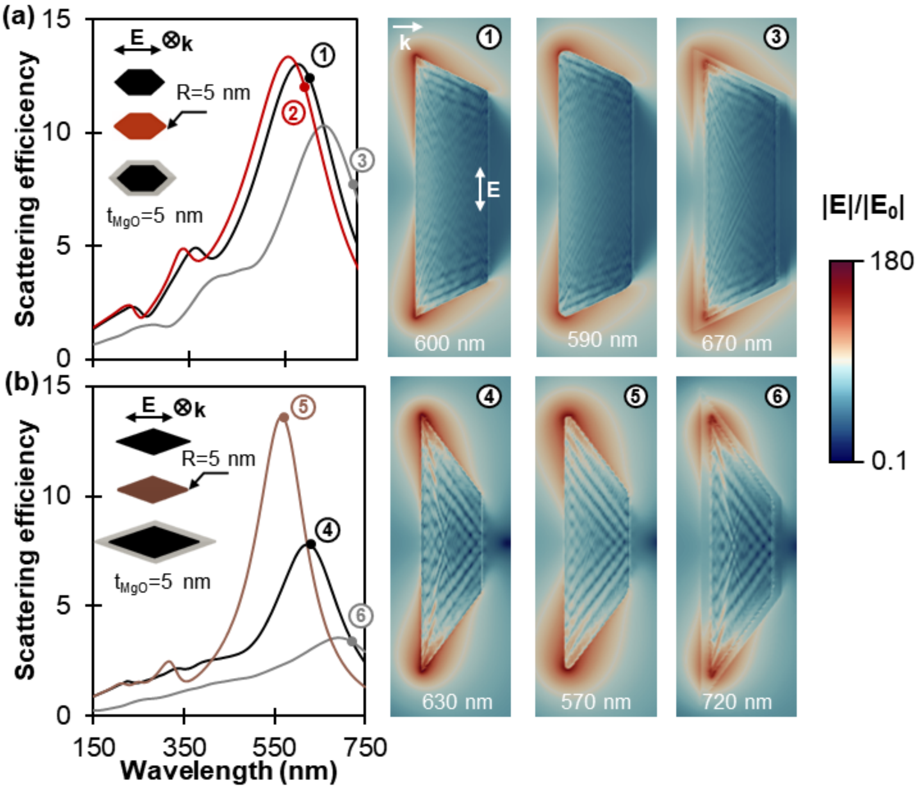

Figure S17. Effect of MgO and tip rounding on the far-field and near-field response. Scattering efficiency and near-field maps for longitudinal polarization for (a) sharp Mg (black), rounded Mg (red), and sharp Mg@MgO (grey) taco NPs, and (b) sharp Mg (black), rounded Mg (brown), and sharp Mg@MgO (grey) $(11\bar{2}4)$ kite NPs. The coloured circles in each scattering spectrum indicate the wavelength position of the maximum field enhancement on the surface of the NP which corresponds to the field maps. Electric field enhancement $|E|/|E_0|$ is plotted in logarithmic scale. NP length, L = 200 nm.

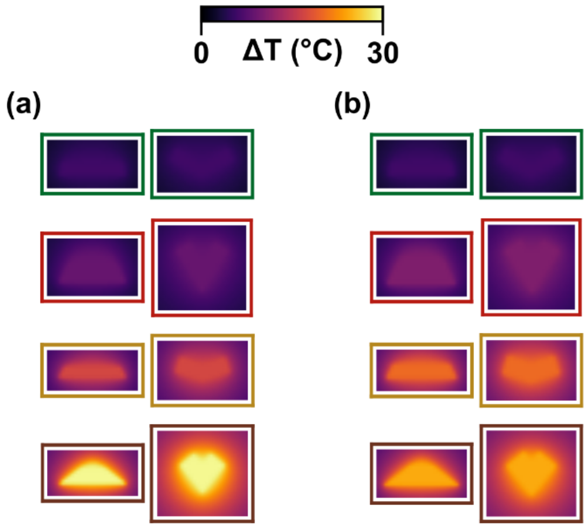

Figure S18. Numerically obtained photothermal response of bare and 5nm MgO-coated NPs. Temperature maps of the two symmetry planes of the shapes shown in colour coded boxes for (a) bare and (b) MgO-coated (t = 5 nm) $(10\bar{1}1)$ tent (green box), $(10\bar{1}3)$ taco (red), $(11\bar{2}1)$ kite (yellow), and $(11\bar{2}4)$ kite (brown) shapes. NP length, L = 200 nm.

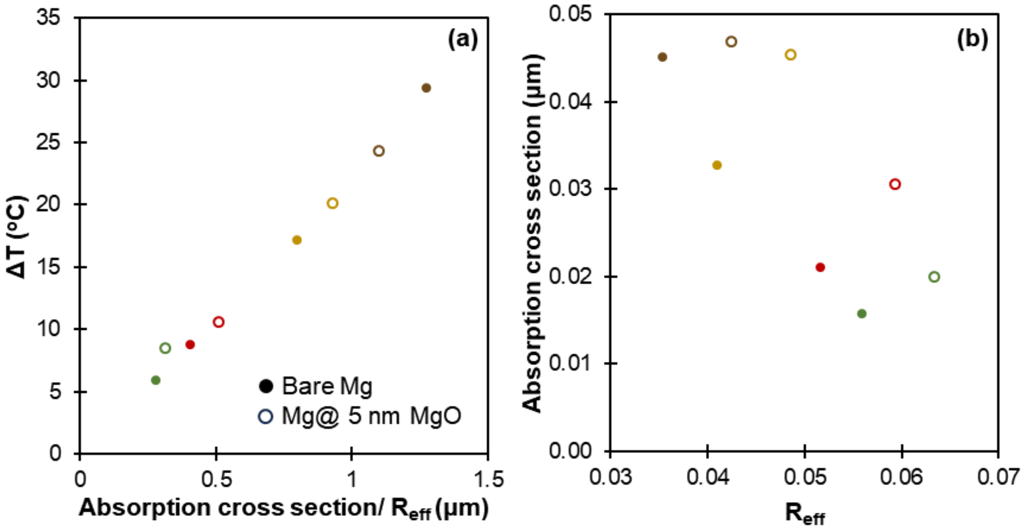

Figure S19. Photothermal response of bare and MgO-coated NPs of various shapes. (a) Plasmon-induced temperature rise as a function of the ratio absorption cross section to effective radius and (b) absorption cross section as a function of the effective radius for the studied Mg NP shapes without (disks) and with (circles) a 5 nm MgO shell. Shape colour coding: tent (green), taco (red), $(11\bar{2}1)$ kite (yellow), and $(11\bar{2}4)$ kite (brown). NP length, L = 200 nm.

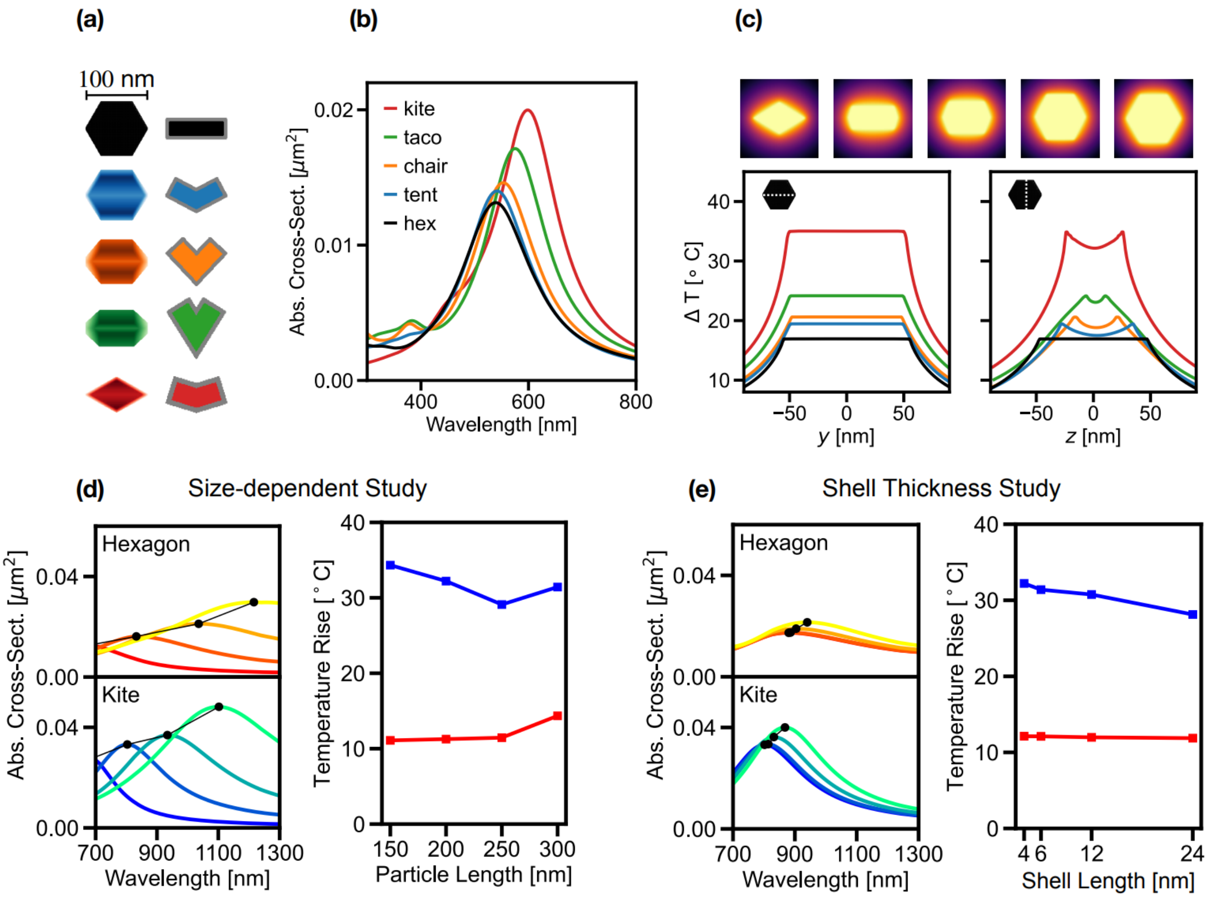

Figure S20: Photothermal response of bare and MgO-coated NPs with varying sizes and oxide thickness. (a) Shapes, (b) absorption cross section, and (c) photoinduced temperature rise for bare Mg NPs of various shapes. (d) Change in absorption cross section and photoinduced temperature rise for a hexagonal prism and $(11\bar{2}1)$ kite of varying sizes (L = 150, 200, 250 and 300 nm). (e) Change in absorption cross section and photoinduced temperature rise for a hexagonal prism and $(11\bar{2}1)$ kite with varying oxide layer thickness (4, 6, 12 and 24 nm).

REFRENCES

(1) Lautar, A. K.; Kopač, D.; Rejec, T.; Bančič, T.; Dominko, R. Morphology Evolution of Magnesium Facets: DFT and KMC Simulations. *Phys. Chem. Chem. Phys.* **2019**, *21* (5), 2434–2442.

(2) Kaminsky, W. WinXMorph (University of Washington, Seattle, 2013) http://cad4.cpac.washington.edu/WinXMorphHome/WinXMorph.htm.
